# Synthesis of racemic and chiral BEDT-TTF derivatives possessing hydroxy groups and their achiral and chiral charge transfer complexes

**DOI:** 10.3762/bjoc.11.172

**Published:** 2015-09-08

**Authors:** Sara Jane Krivickas, Chiho Hashimoto, Junya Yoshida, Akira Ueda, Kazuyuki Takahashi, John D Wallis, Hatsumi Mori

**Affiliations:** 1 The Institute for Solid State Physics, the University of Tokyo, 5-1-5 Kashiwanoha, Kashiwa, Chiba, 277-8581, Japan; 2 The University of Adelaide, Adelaide, South Australia, 5005 Australia; 3 Department of Chemistry, Graduate School of Science, Kobe University, Kobe, Hyogo 657–8501, Japan; 4 School of Science and Technology, Nottingham Trent University, Clifton Lane, Nottingham, NG11 8NS, UK

**Keywords:** BEDT-TTF, chiral molecular crystal, hydrogen bonding, hydroxy group, molecular conductors

## Abstract

Chiral molecular crystals built up by chiral molecules without inversion centers have attracted much interest owing to their versatile functionalities related to optical, magnetic, and electrical properties. However, there is a difficulty in chiral crystal growth due to the lack of symmetry. Therefore, we made the molecular design to introduce intermolecular hydrogen bonds in chiral crystals. Racemic and enantiopure bis(ethylenedithio)tetrathiafulvalene (BEDT-TTF) derivatives possessing hydroxymethyl groups as the source of hydrogen bonds were designed. The novel racemic *trans-vic*-(hydroxymethyl)(methyl)-BEDT-TTF **1**, and racemic and enantiopure *trans-vic*-bis(hydroxymethyl)-BEDT-TTF **2** were synthesized. Moreover, the preparations, crystal structure analyses, and electrical resistivity measurements of the novel achiral charge transfer salt θ^21^-[(*S,S*)-**2**]_3_[(*R,R*)-**2**]_3_(ClO_4_)_2_ and the chiral salt α’-[(*R,R*)-**2**]ClO_4_(H_2_O) were carried out. In the former θ^21^-[(*S,S*)-**2**]_3_[(*R,R*)-**2**]_3_(ClO_4_)_2_, there are two sets of three crystallographically independent donor molecules [(*S,S*)-**2**]_2_[(*R,R*)-**2**] in a unit cell, where the two sets are related by an inversion center. The latter α’-[(*R,R*)-**2**]ClO_4_(H_2_O) is the chiral salt with included solvent H_2_O, which is not isostructural with the reported chiral salt α’-[(*S,S*)-**2**]ClO_4_ without H_2_O, but has a similar donor arrangement. According to the molecular design by introduction of hydroxy groups and a ClO_4_^−^ anion, many intermediate-strength intermolecular hydrogen bonds (2.6–3.0 Å) were observed in these crystals between electron donor molecules, anions, and included H_2_O solvent, which improve the crystallinity and facilitate the extraction of physical properties. Both salts are semiconductors with relatively low resistivities at room temperature and activation energies of 1.2 ohm cm with *E*_a_ = 86 meV for θ^21^-[(*S,S*)-**2**]_3_[(*R,R*)-**2**]_3_(ClO_4_)_2_ and 0.6 ohm cm with *E*_a_ = 140 meV for α'-[(*R,R*)-**2**]_2_ClO_4_(H_2_O), respectively. The variety of donor arrangements, θ^21^ and two kinds of α’-types, and their electrical conductivities of charge transfer complexes based upon the racemic and enantiopure (*S,S*)-**2**, and (*R,R*)-**2** donors originates not only from the chirality, but also the introduced intermolecular hydrogen bonds involving the hydroxymethyl groups, perchlorate anion, and the included solvent H_2_O.

## Introduction

The chiral crystals without an inversion center have attracted much interest recently. Non-centrosymmetric crystals can exhibit a variety of physical properties related to their crystal class [1]; optical and magneto-optical phenomena such as second-harmonic generation, Faraday and Kerr effects, magneto-chiral dichroism, and electrical and magneto-electrical phenomena such as piezoelectricity, pyroelectricity, ferroelectricity and electrical magnetochiral anisotropy. Rikken et al. observed magneto-chiral dichroism in a europium complex [2–3] and also electrical magneto-chiral anisotropy in carbon nanotubes where small changes in the resistance of the chiral carbon nanotubes in a magnetic field were observed between enantiomers [4–6].

**Figure 1:**
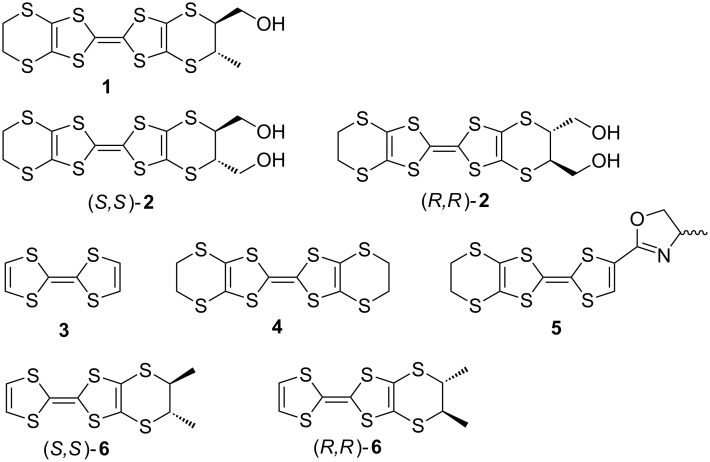
Molecular structures of *trans-vic-*(hydroxymethyl)(methyl)-BEDT-TTF (**1**), *trans-vic-*bis(hydroxymethyl)-BEDT-TTF (**2**), TTF (**3**), BEDT-TTF (**4**), EDT-TTF-methyl-oxazoline **5**, and *trans-*dimethyl-EDT-TTF (**6**).

One method of constructing chiral crystals is through the use of chiral molecules as building blocks. Tetrathiafulvalene derivatives such as TTF (**3**) and BEDT-TTF (**4**) have been investigated considerably due to their ability to form radical cation salts with interesting conductive and magnetic properties (Figure 1). The influence of chirality in the TTF molecules on the crystal structure and physical properties has been shown by Avarvari’s (*R*)-, (*S*)- and racemic (±)-(ethylenedithio(tetrathiafulvalene)methyloxazoline)_2_X, (**5**_2_X, X = AsF_6_ [7], PF_6_ [8]) where disorder within the racemic salt leads to lower conductivity. Recently, Pop et al., have also reported electrical magnetochiral anisotropy in chiral molecular conductors, (*R,R*)- and (*S,S*)-[dimethyl-(ethylenedithio)tetrathiafulvalene]_2_ClO_4_ (**6**_2_ClO_4_) [9–10].

Although there is a relatively large number of chiral TTF derivatives, only a few conducting properties for chiral cation salts have been so far reported, for those based upon tetramethyl-**4** [11–15], **5** [7–8], **6** [9–10], and X-dimethyl-(ethylenedithio)tetrathiafulvalene (X = ethylenedithio [16–17], ethylenedioxy [18], and pyrazino [19]), due to the difficulty of chiral-crystal growth. In order to improve the crystallinity, the inclusion of hydroxy groups in the BEDT-TTF molecule has been postulated to produce hydrogen bonding interactions between electron-donor molecules, electron-acceptor molecules, and anions in the subsequent radical cation salts [20–22]. This may lead to improved order in the crystalline state, which in turn may help the observation of physical properties of the salts. Previously, the synthesis of racemic*-***2** [21–22], the preliminary synthesis of enantiopure (*R,R*)- and (*S,S*)-**2,** and the preparation, and crystal structure of the radical cation salt α’-[(*S*,*S*)-**2**]_2_ClO_4_ [22] have been reported. In this article, we report the syntheses of novel racemic-**1** and enantiopure (*R,R*)- and (*S,S*)-**2** possessing one or two hydroxymethyl groups, and the preparations, crystal structures, and electrical resistivities of the achiral charge transfer complex θ^21^-[(*S,S*)-**2**]_3_[(*R,R*)-**2**]_3_(ClO_4_)_2_ and the chiral complex α’-[(*R,R*)-**2**]_2_ClO_4_(H_2_O), in comparison with those of α’-[(*S*,*S*)-**2**]_2_ClO_4_. The effects of introducing hydrogen bonds between hydroxymethyl groups of donors and ClO_4_^−^ anions in charge transfer complexes are also discussed.

## Results and Discussion

### Syntheses of racemic-**1**, enantiopure (*S*,*S*)- and (*R,R*)-**2** and evaluation of their electrochemical properties

**Scheme 1:**
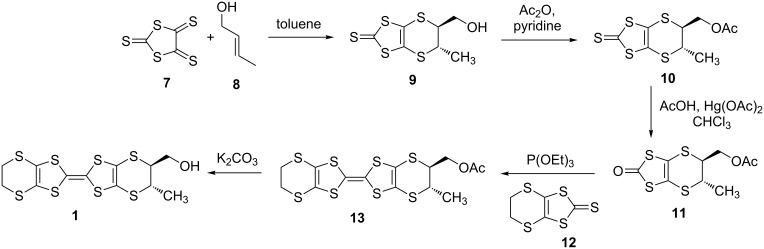
Synthesis of donor *trans-***1**.

The synthesis of the racemic *trans-vic-*(hydroxymethyl)(methyl)-BEDT-TTF (**1**) was performed in a similar manner to racemic **2** [22]. The *trans*-alkene **8** was reacted with trithione **7** under standard Diels–Alder cycloaddition conditions in refluxing toluene to afford a mixture of the *trans*-(*S,S*)- and (*R,R*)-**9** in 56% yield (Scheme 1). The purchased alkene contained a small amount of the *cis*-isomer (*trans*-form:*cis*-form 96:4), but the *cis*-product can be removed by simple recrystallization of the thione **9** from hexane/dichloromethane. The racemic donor **1** could then be synthesized following procedures whereby the alcohol functionality is protected as acetate, **10**, the thione **10** is then converted to the oxo-analogue **11** using mercuric acetate and acetic acid in chloroform. Oxo compound **11** was then cross-coupled with 1.2 equivalents of thione **12** in triethyl phosphite to afford the racemic protected donor **13** in reasonable yield (37%). Basic hydrolysis of the acetyl protecting group afforded the racemic donor **1** in an 81% yield. The syntheses of enatiopure donors **1** and the preparations of their charge transfer complexes are under way.

**Scheme 2:**
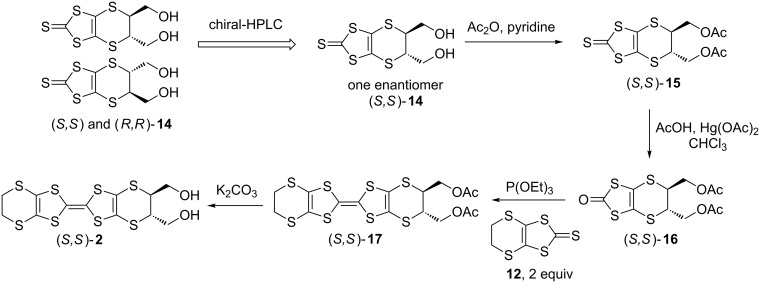
Synthesis of enantiopure donor (*S,S*)-**2**.

Moreover, enantiopure (*S,S*)-**2** and (*R,R*)-**2** were also synthesized as shown in Scheme 2. Chiral HPLC was performed using a JAIGEL-OA7500 column on a JAI LC-908 recycling preparative system using the solvent system methanol/water 7:3 to separate (*S,S*)- and (*R,R*)-**14**. The obtained dihydroxy-thione (*S,S*)-**14** was protected as a diacetate to give (*S,S*)-**15**, which was converted to the oxo-form (*S,S*)-**16**, and coupled with 2 equivalents of **12** to give (*S,S*)-**17**. Deprotection under basic conditions afforded enantiopure (*S,S*)-**2**. The other enantiomer (*R,R*)-**2** was synthesized in the same manner.

The cyclic voltammetry measurement on racemic-**1** indicated the first and second oxidation potentials (*E*^1^_1/2_, *E*^2^_1/2_) and their difference Δ*E* (= *E*^2^_1/2_ − *E*^1^_1/2_) to be 0.52, 0.83, and 0.31 V by utilizing glassy carbon as working electode with 0.1 M tetrabutylammonium perchlorate in benzonitrile. These potentials are similar to those of (*S,S*)- and (*R,R*)-**2** with *E*^1^_1/2_, *E*^2^_1/2_, and Δ*E* of 0.52, 0.80, and 0.28 V, respectively.

**Preparations of single crystals for achiral charge transfer salt** θ**^21^-[(*S,S*)-2]_3_[(*R,R*)-2]_3_(ClO_4_)_2 _and chiral charge transfer salt α’-[(*R,R*)-2]_2_ClO_4_(H_2_O).** The single brown plate crystals of θ^21^-[(*S,S*)-**2**]_3_[(*R,R*)-**2**]_3_(ClO_4_)_2_ were grown by the oxidation of the racemic donor **2** (7 mg) in the presence of tetrabutylammonium perchlorate (44 mg) in dichloromethane (24 mL) at room temperature under a constant current of 0.5 μA under a N_2_ atmosphere during the course of 6 days. The other chiral brown plate crystal of α’-[(*R,R*)-**2**]_2_ClO_4_(H_2_O) was prepared electrochemically by utilizing (*R,R*)-**2** (5 mg) and tetrabutylammonium perchlorate (40 mg) in dichloromethane (9 mL) at 0.5 μA for 5 days.

**Figure 2:**
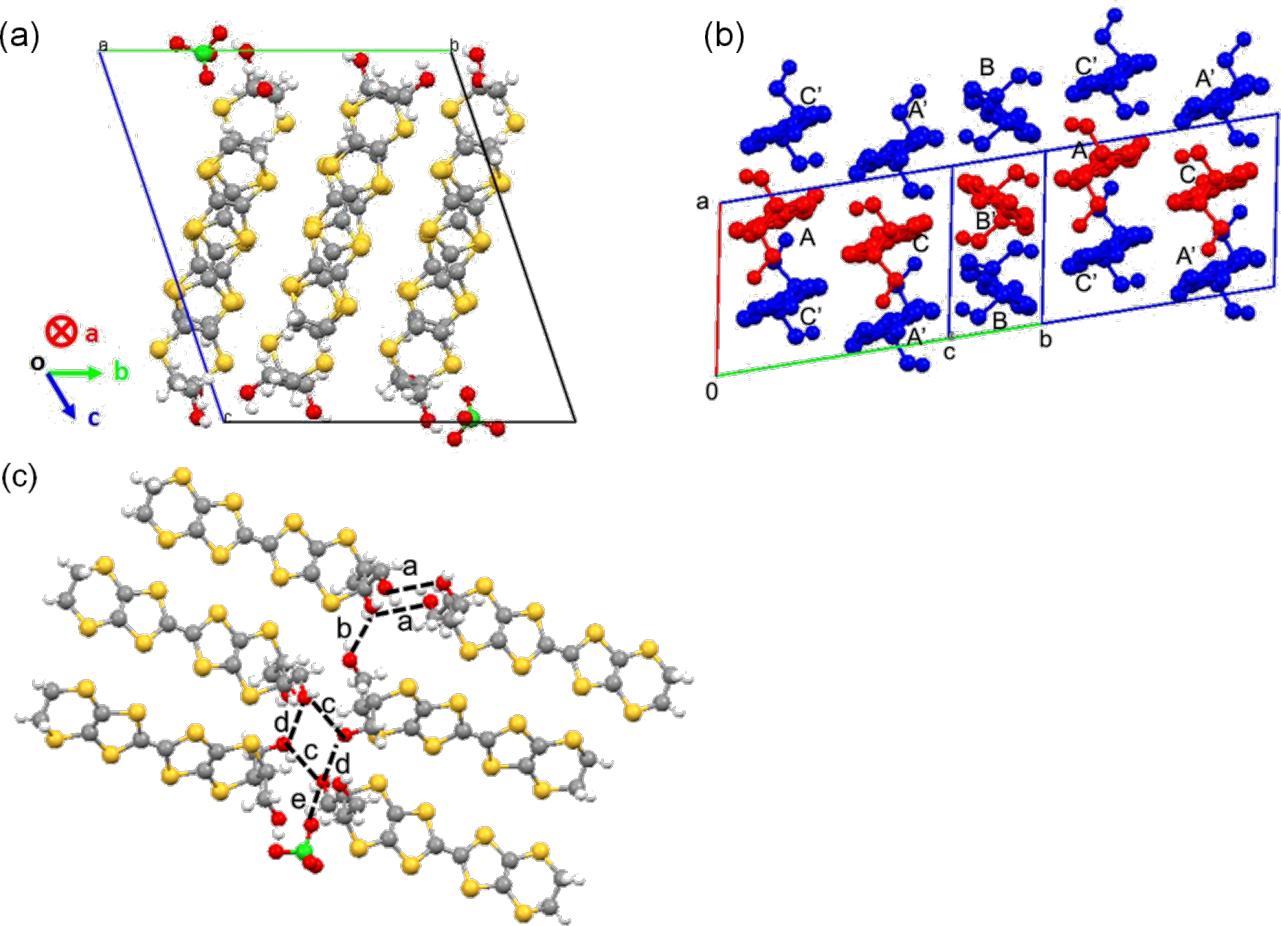
(a) Crystal structure, (b) θ^21^-type donor arrangement of molecules A and A’ [(*S,S*) and (*R,R*)-**2** indicated by red and blue with charge of +0.59(8)], B and B’ [(*R,R*) and (*S,S*)-**2**, blue and red with +0.23(7)], and C and C’ [(*S,S*) and (*R,R*)-**2**, red and blue with +0.18(8)], and (c) O···O hydrogen bond contacts of achital charge transfer salt θ^21^-[(*S,S*)-**2**]_3_[(*R,R*)-**2**]_3_(ClO_4_)_2_; *a* = 2.89(1), *b* = 2.90(1), *c* = 2.879(8), *d* = 2.837(8), *e* = 2.94(1) Å.

**Crystal structures of achiral charge transfer salt θ^21^-[(*S,S*)-2]_3_[(*R,R*)-2]_3_(ClO_4_)_2_ and chiral charge transfer salt α’-[(*R,R*)-2]_2_ClO_4_(H_2_O).** The crystal structure of θ^21^-[(*S,S*)-**2**]_3_[(*R,R*)-**2**]_3_(ClO_4_)_2_ is depicted in Figure 2. The lattice parameters are listed in Table S1 in Supporting Information File 1. The crystallographically independent molecules are two (*S*,*S*)-**2** (molecules A and C indicated by red in Figure 2b), one (*R,R*)-**2** (molecules B indicated by blue), and one ClO_4_^−^ anion. The unit cell contains six donor molecules, consisting of three (*S*,*S*)-**2** (molecules A, B’, and C) and three (*R,R*)-**2** (molecules A’, B and C’), and two ClO_4_^−^ anions. Molecules in a pair, X and X’ (X = A, B, and C) are related by an inversion center, so that the space group is centrosymmetric *P*-1 (No. 2). The donor arrangement is the θ^21^-type, where transvers inclination pattern is ++−++−... (+ and − represent upward and downward slopes, respectively), whereas the usual θ^11^-type has the +−+−+−... pattern [23]. In every (+) and (−)-stacking column, the alternate (*R,R*)–(*S,S*)–(*R,R*)–(*S,S*)- chiral donors indicated by blue–red–blue–red are stacked such as C’AC’A..., A’CA’C..., or BB’BB’... in head-to-tail fashion (Figure 2b). Moreover, the molecules with the same chirality, (*R,R*) or (*S,S*), are arranged side-by-side along the *b*-axis. The charge of each molecule is estimated by bond analyses; as shown in Table S2 (Supporting Information File 1) [24], the charges of molecules A, B, and C are +0.59(8), +0.23(7), and +0.18(8), respectively. In the (+) stacking column, the charge rich A (+0.59(8)) and charge poor C (+0.18(8)) stack, whereas B with the medium charge (+0.23(7)) is arranged in the (−)-column, constructing the appropriate charge balance. The introduced hydroxymethyl groups are set to axial positions for donors A, B, and C (Figure 2b). Following the molecular design, many intermediate-strength intermolecular O···O hydrogen bonds (<2.6–3.0 Å) between hydroxymethyl groups in the donors (*a* = 2.89(1), *b* = 2.90(1), *c* = 2.879(8), and *d* = 2.837(8) Å in Figure 2c), and between a hydroxymethyl group in the donor and a ClO_4_^−^ anion (e = 2.94(1) Å) were observed, presumably helping the crystallinity of the complex based upon chiral molecules.

**Figure 3:**
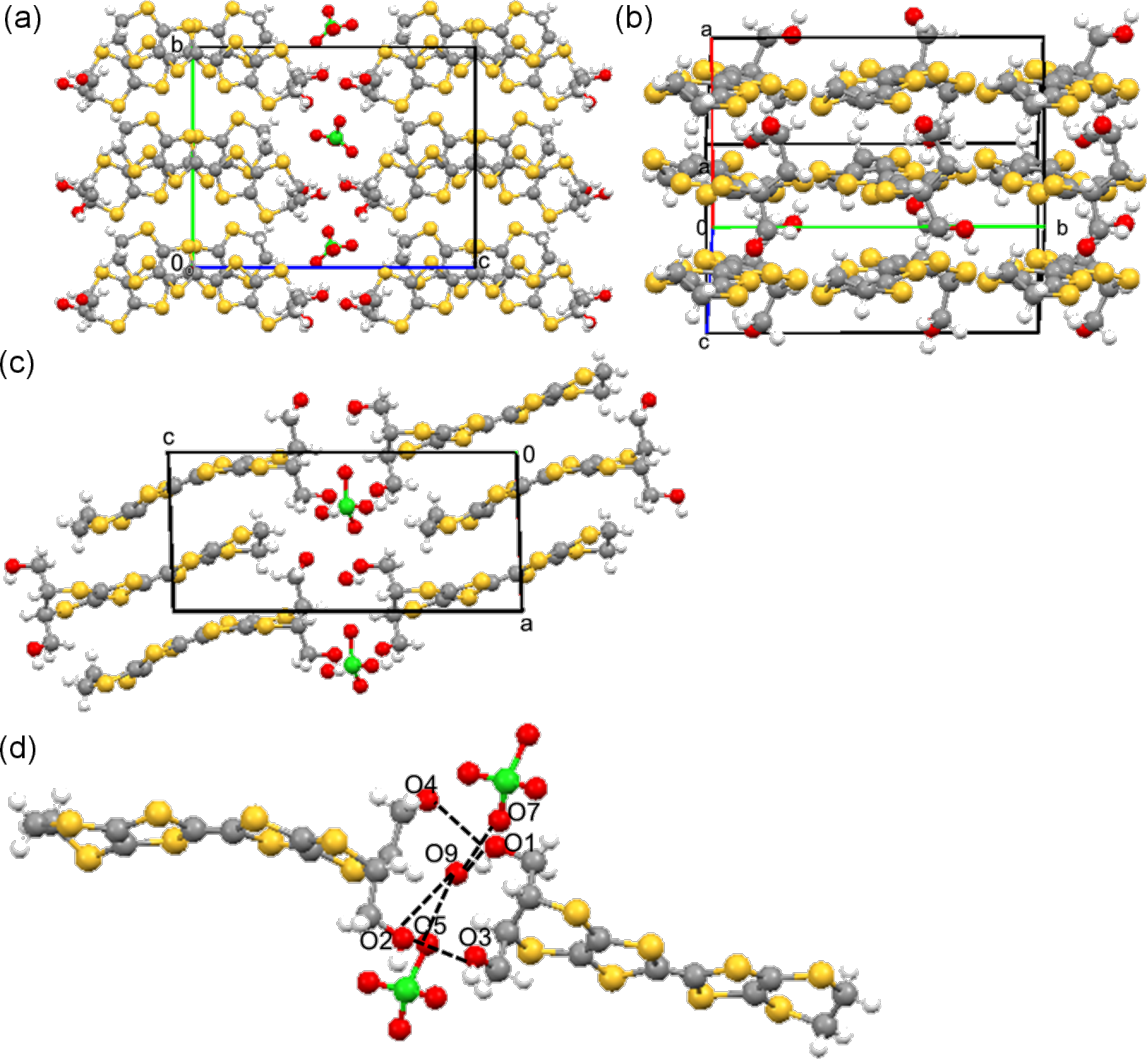
Crystal structure (a) viewed along the *a*-axis, (b) donor arrangement, (c) viewed along the *b*-axis, and (d) intermolecular hydrogen bond contacts for chiral charge transfer salt α'-[(*R,R*)-**2**]_2_ClO_4_(H_2_O). The calculated hydrogen bonds are as follows; *r*(O1···O4) = 2.67(3), *r*(O1···O9) = 3.00(3), *r*(O2···O3) = 2.67(2), *r*(O2···O9) = 2.85(3), *r*(O5···O9) = 2.62(6), and *r*(O7···O9) = 2.90(6) Å.

The crystal structure of the chiral salt α’-[(*R, R*)*-***2**]_2_ClO_4_(H_2_O) is shown in Figure 3. The crystallographically independent molecules are two (*R,R*)*-***2** donors, one ClO_4_^−^ anion, and one H_2_O molecule as an included solvent. There are four donors, two anions, and two H_2_O solvents in the unit cell. The enantiopure (*R,R*)*-***2** donors stack in a head-to-tail manner and twisted with respect to each other along the *a*-axis, namely the α’-type donor arrangement [25].

The hydroxymethyl groups project from the molecular BEDT-TTF plane in axial positions (Figure 3c). According to the molecular design, the intermolecular moderate hydrogen bonds between the oxygen atoms in hydroxymethyl groups of (*R,R*)-**2** donors are observed such as *r*(O1···O4) = 2.67(3) and *r*(O2···O3) = 2.67(2) Å (Figure 3d). The other hydrogen bonds are found between the oxygen atoms of either included H_2_O solvent and hydroxymethyl groups such as *r*(O1···O9) = 3.00(3) and *r*(O2···O9) = 2.85(3) Å, or of a solvent H_2_O and an anion ClO_4_^−^ such as *r*(O5···O9) = 2.62(6) and *r*(O7···O9) = 2.90(6) Å. These hydrogen bonds contribute to forming this chiral crystal.

This chiral crystal α’-[(*R,R*)*-***2**]_2_ClO_4_(H_2_O) is not isostructural to the other enantiopure crystal α'-[(*S,S*)-**2**]_2_ClO_4_, previously reported (Table S1, Supporting Information File 1) [22]. The crystallographically independent donors are two for α’-[(*R,R*)*-***5**]_2_ClO_4_(H_2_O) in the space group *P*2_1_ (No. 4), but one for α'-[(*S,S*)-**5**]_2_ClO_4_ in *P*2 (No. 3). Although this α’-[(*R,R*)*-***2**]_2_ClO_4_(H_2_O) crystal includes a H_2_O solvent molecule and α'-[(*S,S*)-**2**]_2_ClO_4_ does not in the same preparation conditions, both salts have the similar α’-type donor arrangements.

**Figure 4:**
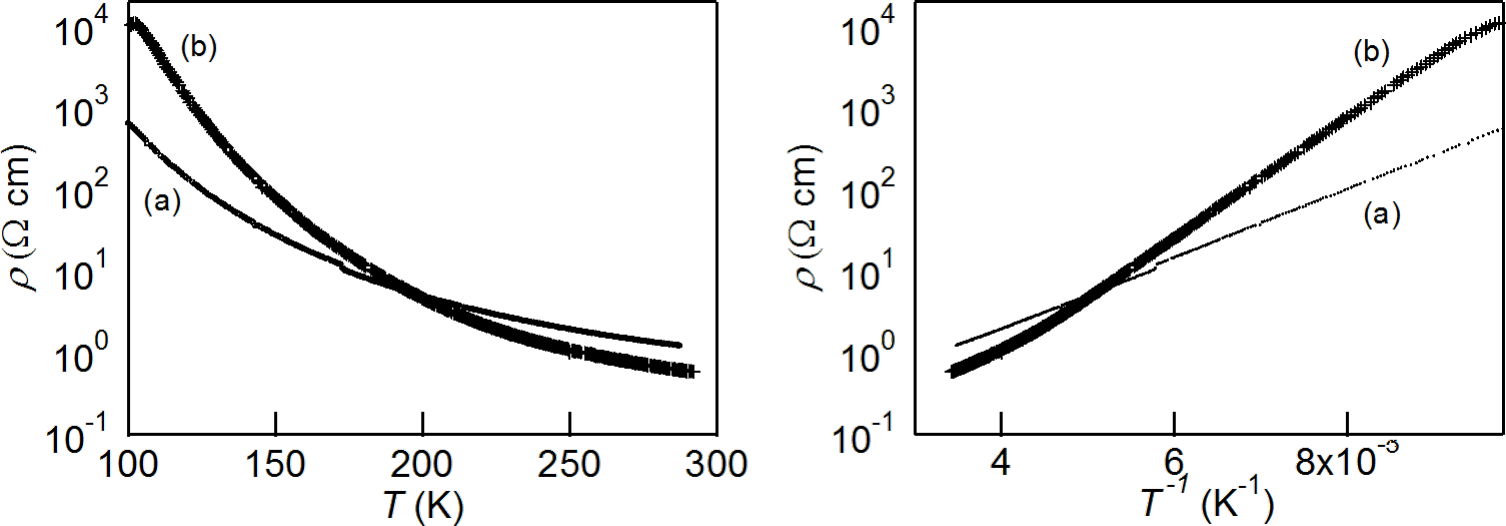
Temperature dependences of electrical resistivities for (a) achiral charge transfer salt θ^21^-[(*S,S*)-**2**]_3_[(*R,R*)-**2**]_3_(ClO_4_)_2_ and (b) chiral salt α'-[(*R,R*)-**2**]_2_ClO_4_(H_2_O).

**Electrical resistivities for the achiral charge transfer salt θ^21^-[(*S,S*)-2]_3_[(*R,R*)-2]_3_(ClO_4_)_2_ and the chiral salt α’-[(*R,R*)-2]_2_ClO_4_(H_2_O).** Temperature dependences of electrical resistivites for the achiral charge transfer salt θ^21^-[(*S,S*)-**2**]_3_[(*R,R*)-**2**]_3_(ClO_4_)_2_ and the chiral salt α'-[(*R,R*)-**2**]_2_ClO_4_(H_2_O) are shown in Figure 4. The resistivities at room temperature are very similar, 1.2 and 0.6 ohm cm for θ^21^-[(*S*,*S*)-**2**]_3_[(*R,R*)-**2**]_3_(ClO_4_)_2_ and α'-[(*R,R*)-**2**]_2_ClO_4_(H_2_O), respectively. Both salts show semiconducting behaviour and the activation energy of θ^21^-[(*S*,*S*)-**2**]_3_[(*R,R*)-**2**]_3_(ClO_4_)_2_ is *E*_a_ = 86 meV which is lower than that of α'-[(*R,R*)-**2**]_2_ClO_4_(H_2_O) which has *E*_a_ = 140 meV.

## Conclusion

In summary, we have synthesized redox-active racemic and enantiopure donors of BEDT-TTF derivatives containing one or two hydroxymethyl groups, the novel racemic *trans*-*vic-*(hydroxymethyl)(methyl)-BEDT-TTF **1** and enantiopure *vic*-bis(hydroxymethyl)-BEDT-TTF **2**. By successful molecular design to introduce intermolecular hydrogen bonds, the achiral charge transfer salt θ^21^-[(*S,S*)-**2**]_3_[(*R,R*)-**2**]_3_(ClO_4_)_2_ and the chiral salt α’-[(*R, R*)-**2**]ClO_4_(H_2_O) could be obtained, and their crystal structure analyses and measurements of electrical resistivities were performed. In the racemic complex θ^21^-[(*S,S*)-**2**]_3_[(*R,R*)-**2**]_3_(ClO_4_)_2_*,* (*S,S*)-**2** and (*R,R*)-**2** donors stack alternately along the *a*-axis and the same chiral (*S,S*)-**2** (or (*R,R*)-**2**) donors are arranged in the side-by-side interaction to construct the stripe chirality order. The latter chiral salt α’-[(*R,R*)-**2**]ClO_4_(H_2_O) is not isostructural with α’-[(*S,S*)-**2**]ClO_4_ without H_2_O, but has a similar α’-type donor arrangement. According to the molecular design, both crystals have many moderate-strength hydrogen bonds of 2.6–3.0 Å between donor molecules, ClO_4_^−^ anions, and H_2_O, which contribute to crystallinity based upon chiral molecules and allow the investigation of physical properties. The promising strategy of chiral crystal growth will lead to the development of the versatile functionalities of molecular chiral crystals.

## Experimental

### General information

The parent racemic-**2** (Figure 1) was synthesized according to the literature [22]. ^1^H NMR (300 MHz) and ^13^C NMR (75 MHz) spectra were measured with a JEOL JNM-AL300 spectrometer with CDCl_3_ as solvent using Me_4_Si or residual solvent as an internal standard. Cyclic voltammetry (CV) measurements were performed on an ALS 610DB electrochemical analyzer in benzonitrile containing 0.1 M tetrabutylammonium perchlorate (working electrode: Pt, counter electrode: Pt wire, reference electrode: saturated calomel electrode (SCE)) [22]. EI-mass spectra were obtained with a JEOL JMS-AX500 spectrometer. X-ray crystallographic measurements were made on a Rigaku AFC-7R diffractometer (Mo Kα, λ = 0.71073 Å). The crystal structures were solved by direct methods and refined with full-matrix least-squares technique using Crystal Structure (ver. 4.0.1, Rigaku Co. and Rigaku Americas Co.). Anisotropic thermal parameters were applied to all non-hydrogen atoms. The hydrogen atoms were generated geometrically (C–H = 0.960 Å). The direct current electrical conductivity measurement was made by the conventional four-probe method using carbon paste and gold wires.

### Synthesis of racemic-1

***trans*-5-(Hydroxymethyl)-6-methyl-5,6-dihydro[1,3]dithiolo[4,5-*b*][1,4]dithiine-2-thione (9).** A solution of trithione **7** (1 g, 5.1 mmol, 1 equiv) and alkene **8** (0.52 mL, 6.12 mmol, 1.2 equiv, *trans-*form 96%) in toluene (50 mL) was refluxed overnight. The toluene was removed under reduced pressure and the crude material purified by column chromatography (silica gel, 2:1 hexane:ethyl acetate) to afford the *trans*-thione **9** as a brown powder (with small amounts of the *cis* derivative). This material was recrystallized from dichloromethane/hexane to give the *trans*-thione **9** as a brown powder (0.77g, 56%). ^1^H NMR (300 MHz, CDCl_3_) δ 1.55(d, *J* = 6.6 Hz, 3H), 1.98 (dd, *J* = 5.1, 6.6 Hz, 1H), 3.36 (ddd, *J* = 3.9, 6.3, 7.5 Hz, 1H), 3.64 (dq, *J* = 3.9, 6.9 Hz, 1H), 3.86 (m, 2H); ^13^C NMR (75 MHz, CDCl_3_) δ 21.90, 37.07, 49.95, 64.89, 120.20, 120.46, 207.57.

***trans*-5-(Acetoxymethyl)-6-methyl-5,6-dihydro[1,3]dithiolo[4,5-*b*][1,4]dithiine-2-thione (10).** To a solution of thione **9** (482 mg, 1.80 mmol, 1 equiv) in pyridine (5 mL) was added acetic anhydride (202 mg, 1.98 mmol, 1.1 equiv). The reaction mixture was stirred overnight under argon at room temperature. Dichloromethane (50 mL) was added, the organic phase was washed with 1 M HCl (2 × 50 mL) and water (50 mL), dried over MgSO_4_, filtered and reduced in vacuo to give the required product **10** as a brown oil (560 mg, 100%). The material was used in the next step without further purification. ^1^H NMR (300 MHz, CDCl_3_) δ 1.50 (d, *J* = 6.9 Hz, 3H), 2.03 (s, 3H), 3.42 (ddd, *J* = 3.9, 6.6, 7.8 Hz, 1H), 3.50 (dq, *J* = 3.9, 6.9 Hz, 1H), 4.22 (dd, *J* = 7.5, 11.7 Hz, 1H), 4.27 (dd, *J* = 6.0, 11.7 Hz, 1H); ^13^C NMR (75 MHz, CDCl_3_) δ 20.69, 21.82, 37.13, 45.86, 65.66, 119.44, 119.82, 170.33, 207.20.

***trans*-5-(Acetoxymethyl)-6-methyl-5,6-dihydro[1,3]dithiolo[4,5-*b*][1,4]dithiine-2-one (11).** To a solution of thione **10** (560 mg, 1.81 mmol, 1 equiv) in chloroform (30 mL) was added mercuric acetate (843 mg, 2.71 mmol, 1.5 equiv) and acetic acid (5 mL). The reaction mixture was stirred for 3 hours at room temperature. The solution was filtered, washed with sat. NaHCO_3_ (2 × 50 mL) and water (50 mL), dried over MgSO_4_, filtered and reduced in vacuo to give the oxo compound **11** (480 mg, 90%) as a yellow solid. ^1^H NMR (300 MHz, CDCl_3_) δ 1.53 (d, *J* = 6.6 Hz, 3H), 2.03 (s, 3H), 3.40 (ddd, *J* = 3.9, 6.3, 7.2 Hz, 1H), 3.46 (dq, *J* = 3.9, 6.6 Hz, 1H), 4.28 (m, 2H); ^13^C NMR (75 MHz, CDCl_3_) δ 20.73, 21.80, 38.50, 47.59, 65.82, 110.40, 110.61, 116.36, 170.43.

***trans*-*vic*-Acetoxymethyl-methyl-BEDT-TTF (13).** A solution of thione **12** (439 mg, 1.96 mmol, 1.2 equiv) and the oxo-compound **11** (480 mg, 1.63 mmol, 1 equiv) in triethyl phosphite (10 mL) was stirred overnight at 90 °C. After cooling, hexane was added and the precipitate collected by vacuum filtration and purified by column chromatography (silica gel, 4:1 hexane/ethyl acetate) to afford the cross-coupled product **13** as a red-orange solid (280 mg, 37%). ^1^H NMR (300 MHz, CDCl_3_) δ 1.44 (d, *J* = 6.6 Hz, 3H), 2.05 (s, 3H), 3.26 (s, 4H), 3.38 (ddd, *J* = 3.9, 6.3, 7.5 Hz, 1H), 3.43 (dq, *J* = 3.9, 6.6 Hz, 1H), 4.18 (dd, *J* = 7.5, 11.4 Hz, 1H), 4.24 (dd, *J* = 6.3, 11.4 Hz, 1H); ^13^C NMR (75 MHz, CDCl_3_) δ 20.75, 21.58, 30.06, 37.80, 46.56, 65.80, 110.54, 110.77, 110.97, 111.86, 113.69, 170.35.

***trans*-*vic*-Hydroxymethyl-methyl-BEDT-TTF (1).** A solution of the acetyl protected BEDT-TTF **13** (280 mg, 0.596 mmol, 1 equiv) and K_2_CO_3_ (95 mg, 0.894 mmol, 1.5 equiv) in MeOH/H_2_O/THF (5 mL/5 mL/5 mL) was stirred overnight at room temperature under an argon atmosphere. Water (50 mL) and dichloromethane (100 mL) were added, the organic layer separated and washed with water (2 × 50 mL). The organic layer was dried over MgSO_4_, filtered and evaporated to give the crude product that was purified by column chromatography (silica gel, ethyl acetate) to afford the *trans*-racemic BEDT-TTF derivative **1** as an orange powder (210 mg, 81%). ^1^H NMR (300 MHz, CDCl_3_) δ 1.43 (d, *J* = 6.6 Hz, 3H), 1.85 (br s, 1H), 3.22 (s, 4H), 3.24 (m, 1H), 3.46 (dq, *J* = 3.6, 6.6 Hz, 1H), 3.71 (m, 2H); ^13^C NMR (75 MHz, DMSO-*d*_6_) δ 21.29, 29.58, 37.39, 50.37, 63.98, 110.03, 110.11, 110.44, 111.62, 112.93, MS *m*/*z*: [M – OH]^+^ calcd for C_12_H_11_S_8_, 411; found, 410.8627.

### Synthesis of (*S,S*)-**2**

**(*S,S*)-*trans*-5,6-Bis(acetyloxymethyl)-5,6-dihydro-1,3-dithiolo[4,5-*b*]-1,4-dithiin-2-thione ((*S,S*)-15).** A solution of the dihydroxythione (*S,S*)-**14** (25 mg, 0.088 mmol, 1 equiv) in pyridine (1 mL) and acetic anhydride (0.02 mL, 1.96 mmol, 20 equiv) was stirred at room temperature overnight under an argon atmosphere. The reaction mixture was taken up in dichloromethane and washed briefly with 1 M HCl (2 × 50 mL) and water (50 mL). The organic layer was dried over MgSO_4_, filtered and the volatiles removed in vacuo to give the crude enantiopure diacetoxythione (*S,S*)-**15** (32 mg, quantitative). The isolated product was used in the next step without further purification. ^1^H NMR (300 MHz, CDCl_3_) δ 2.12 (s, 6H), 3.79 (m, 2H), 4.32 (dd, *J* = 8.1, 11.4 Hz, 2H), 4.42 (dd, *J* = 6.0, 11.4 Hz, 2H); ^13^C NMR (75 MHz, CDCl_3_) δ 20.69, 40.06, 64.71, 118.79, 170.28, 206.49.

**(*S,S*)-*trans*-5,6-Bis(acetoxymethyl)-5,6-dihydro-1,3-dithiolo[4,5-*b*]-1,4-dithiin-2-one ((*S,S*)-16).** To a solution of thione (*S,S*)-**15** (32 mg, 0.087 mmol, 1 equiv) in chloroform (10 mL) was added mercuric acetate (35 mg, 0.11 mmol, 1.3 equiv) and acetic acid (0.5 mL). The reaction mixture was stirred for 3 hours, after which it was filtered, washed with (sat.) NaHCO_3_ (3 × 50 mL) and water (50 mL). The organic layer was dried over MgSO_4_, filtered and the solvent removed under reduced pressure to afford the oxo compound (*S,S*)-**16** (28 mg (93%). ^1^H NMR (300 MHz, CDCl_3_) δ 2.12 (s, 6H), 3.76 (m, 2H), 4.38 (dd, *J* = 7.5, 11.4 Hz, 2H), 4.45 (dd, *J* = 5.7, 11.4 Hz, 2H); ^13^C NMR (75 MHz, CDCl_3_) δ 20.69, 41.40, 64.84, 109.72, 170.35, 187.57.

**(*S,S*)-*trans-vic-*Bis(acetyloxymethyl)-BEDT-TTF ((*S,S*)-17).** A solution of thione **12** (64 mg, 0.16 mmol, 2 equiv) and oxo compound (*S,S*)-**16** (50 mg, 0.14 mmol, 1 equiv) in triethyl phosphite (2 mL) was stirred at 90 °C for 5 hours. After cooling hexane was added, the precipitate collected and purified by column chromatography (silica gel, hexane/ethyl actetate) to afford the enantiopure cross coupled product (*S,S*)-**17** (34 mg, 47%). ^1^H NMR (300 MHz, CDCl_3_) δ 2.09 (s, 6H), 3.29 (s, 4H), 3.69 (m, 2H), 4.25 (dd, *J* = 7.8, 11.4 Hz, 2H), 4.34 (dd, *J* = 6.0, 11.4 Hz, 2H); ^13^C NMR (75 MHz, CDCl_3_) δ 20.73, 30.17, 40.88, 64.93, 110.43, 113.92, 170.35.

**(*S,S*)-*trans-vic-*Bis(hydroxymethyl)-BEDT-TTF ((*S,S*)-2).** K_2_CO_3_ (27 mg, 0.193 mmol, 3 equiv) was added to a solution of the acetyl protected BEDT-TTF ((*S,S*)-**17**, 34 mg, 0.064 mmol) in MeOH/THF/H_2_O (1 mL/1 mL/1 mL). The reaction mixture was stirred for 3 hours under an argon atmosphere, after which no starting material remained (TLC control). Dichloromethane (100 mL) was added and the organic layer washed with water (2 × 20 mL). The organic layer was dried over MgSO_4_, filtered and the solvent was removed under reduced pressure. The crude material was purified by column chromatography (silica gel, 1:1 hexane/ethyl acetate) to afford the desired product (*S,S*)-**2** (23 mg, 80%) as a red–pink solid. ^1^H NMR (300 MHz, DMSO-*d*_6_) δ 3.34 (s, 4H), 3.58 (m, 4H), 3.71 (m, 2H), 4.44 (m, 2H); ^13^C NMR (75 MHz, DMSO-*d*_6_) δ 30.83, 45.82, 67.40, 111.24, 111.38, 111.97, 114.44.

## Supporting Information

File 1 CD spectra of (*S,S*)-**2** and (*R,R*)-**2**, crystal data for θ^21^-[(*S,S*)-**2**]_3_[(*R,R*)-**2**]_3_(ClO_4_)_2_, α’-[(*R,R*)-**2**]_2_ClO_4_(H_2_O), and α’-[(*S,S*)-**2**]_2_ClO_4_ (Table S1), charge estimation of θ^21^-[(*S,S*)-**2**]_3_[(*R,R*)-**2**]_3_(ClO_4_)_2_ (Table S2, Figure S2), and NMR data (Figures S3-1–S3-18).
