# Supplementary material for: Synthesis of racemic and chiral BEDT-TTF derivatives possessing hydroxy groups and their achiral and chiral charge transfer complexes
Source: Beilstein J Org Chem. 2015 Sep 8;11:1561–9. doi: 10.3762/bjoc.11.172 (PMC4660918; doi:10.3762/bjoc.11.172)
Supplement: File 1 — CD spectra of (S,S)-2 and (R,R)-2, crystal data for θ21-[(S,S)-2]3[(R,R)-2]3(ClO4)2, α’-[(R,R)-2]2ClO4(H2O), and α’-[(S,S)-2]2ClO4 (Table S1), charge estimation of θ21-[(S,S)-2]3[(R,R)-2]3(ClO4)2 (Table S2, Figure S2), and NMR data (Figures S3-1–S3-18). [file Beilstein_J_Org_Chem-11-1561-s001.pdf]

Supporting Information

for

Synthesis of racemic and chiral BEDT-TTF derivatives possessing hydroxy groups and their achiral and chiral charge transfer complexes

Sara J. Krivickas[1,2], Chiho Hashimoto[1], Junya Yoshida[1], Akira Ueda[1], Kazuyuki Takahashi[1,3], John D. Wallis[4] and Hatsumi Mori*[1]

Address: [1]The Institute for Solid State Physics, the University of Tokyo, 5-1-5 Kashiwanoha, Kashiwa, Chiba, 277-8581, Japan, [2]The University of Adelaide, Adelaide, South Australia, 5005 Australia, [3]Department of Chemistry, Graduate School of Science, Kobe University, Kobe, Hyogo 657–8501, Japan, [4]School of Science and Technology, Nottingham Trent University, Clifton Lane, Nottingham, NG11 8NS, UK

Email: Hatsumi Mori - hmori@issp.u-tokyo.ac.jp

* Corresponding author

CD spectra of (*S,S*)-2 and (*R,R*)-2, crystal data for θ^{21}-[(*S,S*)-2]$_3$[(*R,R*)-2]$_3$(ClO_4)$_2$, α'-[(*R,R*)-2]$_2$$ClO_4$($H_2O$), and α'-[(*S,S*)-2]$_2$$ClO_4$ (Table S1), charge estimation of θ^{21}-[(*S,S*)-2]$_3$[(*R,R*)-2]$_3$(ClO_4)$_2$ (Table S2, Figure S2), and NMR data (Figures S3-1–S3-18)

Table of Contents

CD spectra of (*S,S*)-**2** and (*R,R*)-**2** S3

Crystal data for θ^{21}-[(*S,S*)-**2**]$_3$[(*R,R*)-**2**]$_3$(ClO_4)$_2$, α'-[(*R, R*)-**2**]$_2$$ClO_4$($H_2O$), and α'-[(*S, S*)-**2**]$_2$$ClO_4$ S4

Charge estimation of θ^{21}-[(*S,S*)-**2**]$_3$[(*R,R*)-**2**]$_3$(ClO_4)$_2$ S5

NMR data S6

References S24

CD spectra of (*S,S*)-2 and (*R,R*)-2

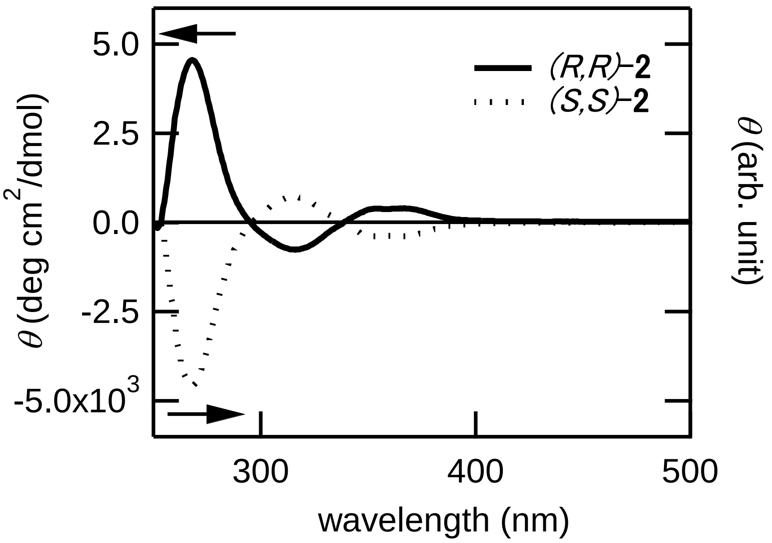

Figure S1: CD spectra of (*R*,*R*)-**2** and (*S*,*S*)-**2**.

Supporting crystal structural data

Table S1: Crystal data for achiral charge transfer salt θ^{21}-[(*S,S*)-**2**]$_3$[(*R,R*)-**2**]$_3$(ClO$_4$)$_2$,, chiral charge transfer salts α'-[(*R,R*)-**2**]$_2$ClO$_4$(H$_2$O), and α'-[(*S,S*)-**2**]$_2$ClO$_4$.

	θ^{21}-[(*S,S*)-**2**]$_3$[(*R,R*)-**2**]$_3$ (ClO$_4$)$_2$,	α'-[(*R,R*)-**2**]$_2$-ClO$_4$(H$_2$O)	α'-[(*S,S*)-**2**]$_2$ClO$_4$
Formula	$C_{72}H_{72}S_{48}O_{20}Cl_2$	$C_{24}H_{26}O_9S_{16}Cl_1$	$C_{24}H_{24}O_8S_{16}Cl_1$
Formula Weight	2867.14	1006.88	988.86
Crystal System	triclinic	monoclinic	monoclinic
Space Group	*P*-1 (#2)	$P2_1$ (#4)	*P*2 (#3)
Temperature / K	293	105	273
λ / Å	0.71073	0.71073	0.71073
a / Å	8.653(3)	7.951(5)	8.112(15)
b / Å	17.321(4)	13.619(7)	6.893(13)
c / Å	19.294(5)	17.492(10)	17.64(3)
α / °	70.334(11)	90	90
β / °	80.611(13)	92.052(8)	91.30(3)
γ / °	81.454(13)	90	90
V / Å^3	2673.0(13)	1892.9(18)	986(3)
Z value	1	2	1
d_{calc} / g cm^{-3}	1.781	1.766	1.665
GOF	1.630	0.970	1.413
*R*1 (I >2.00σ(I))	0.0777	0.0926	0.1152
*wR*2 (All data)	0.1858	0.2949	0.1132
flack parameter	-	0.1(3)	-
# of observations	7517	8581	2489
# of variables	671	431	85
CCDC	1404299	1404300	997838
Reference	This work	This work	S1

Table S2: Charge estimation of the TTF skeleton in θ^{21}-[(*S,S*)-**2**]$_3$[(*R,R*)-**2**]$_3$(ClO_4)$_2$, based on its bond lengths (Figure S2, see below).[S2] Q and Q' represent the tentative and normalized charges where the sum of three charges is set to +1, respectively.
[a]Q = 6.347–7.463*[(b1+b2]/2+(b'1+b'2)/2+(c1+c2]/2+(c'1+c'2)/2-a-(d+d')/2], [b]Q' (molecule A) = [Q'(molecule A) / [(Q'(molecule A)+Q'(molecule B)+Q'(molecule C)]

	molecule A	molecule B	molecule C
a / Å	1.376(7)	1.344(7)	1.341(7)
b1, b2 / Å	1.730(6), 1.744(7)	1.756(5), 1.754(6)	1.748(6), 1.747(7)
b'1, b'2 / Å	1.738(7), 1.740(6)	1.745(6), 1.750(5)	1.750(7), 1.746(6)
c1, c2 / Å	1.749(6), 1.748(5)	1.765(5), 1.750(5)	1.748(6),1.750(5)
c'1, c'2 / Å	1.749(5), 1.739(6)	1.738(5), 1.751(5)	1.752(5), 1.745(6)
d / Å	1.345(9)	1.333(8)	1.338(10)
d' / Å	1.359(10)	1.356(8)	1.329(10)
Q[a]	0.70(8)	0.27(7)	0.21(7)
Q'[b]	0.59(8)	0.23(7)	0.18(8)

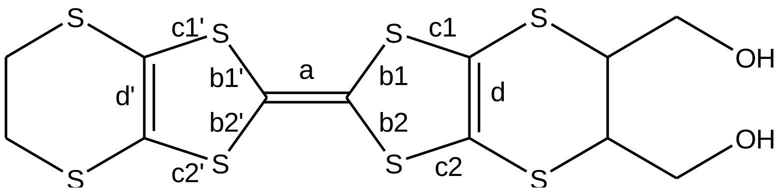

Figure S2: Bond numbering scheme for the TTF skeleton.

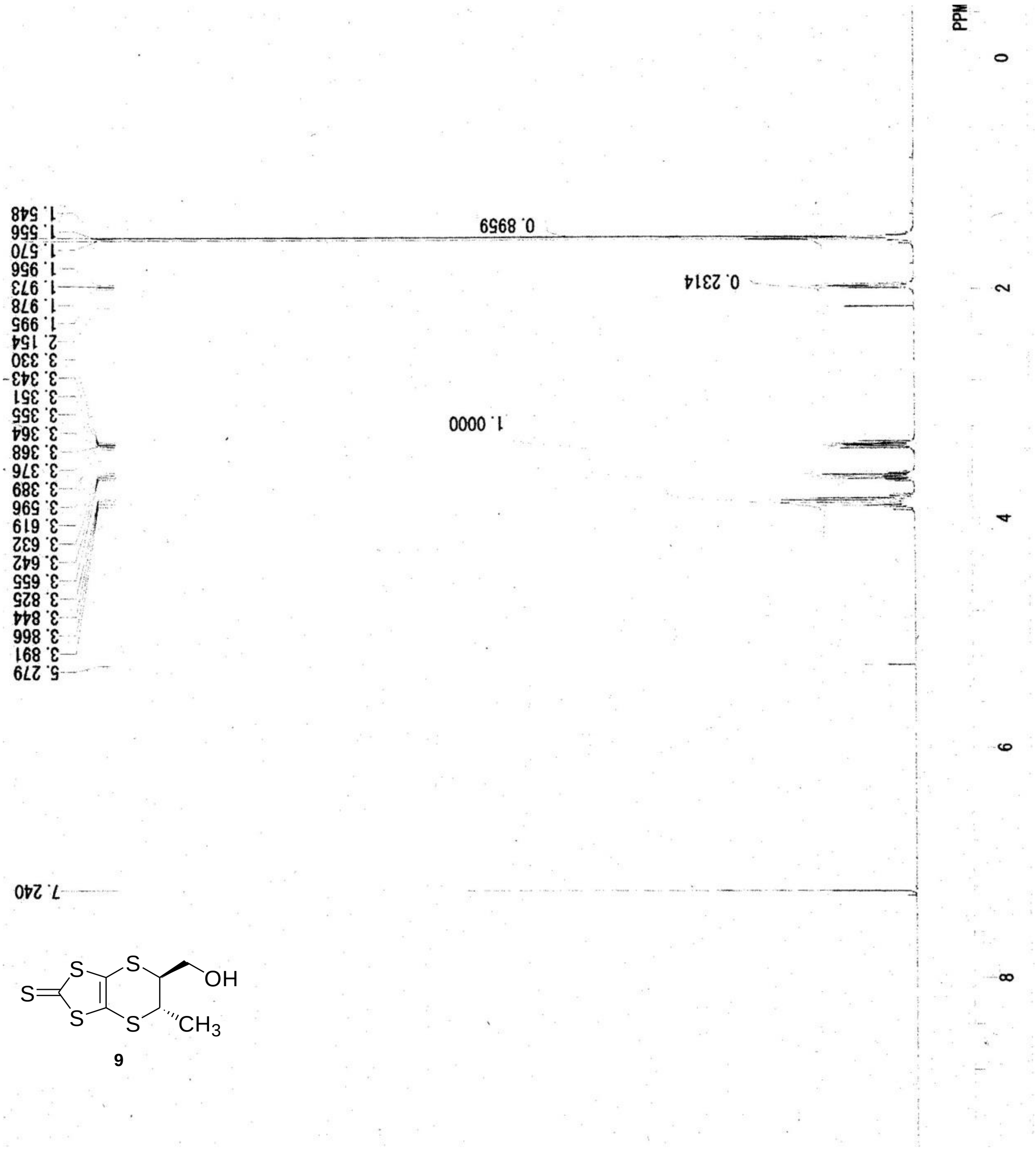

Figure S3-1: ^{1}H NMR (300 MHz, $CDCl_3$) spectrum of compound **9**.

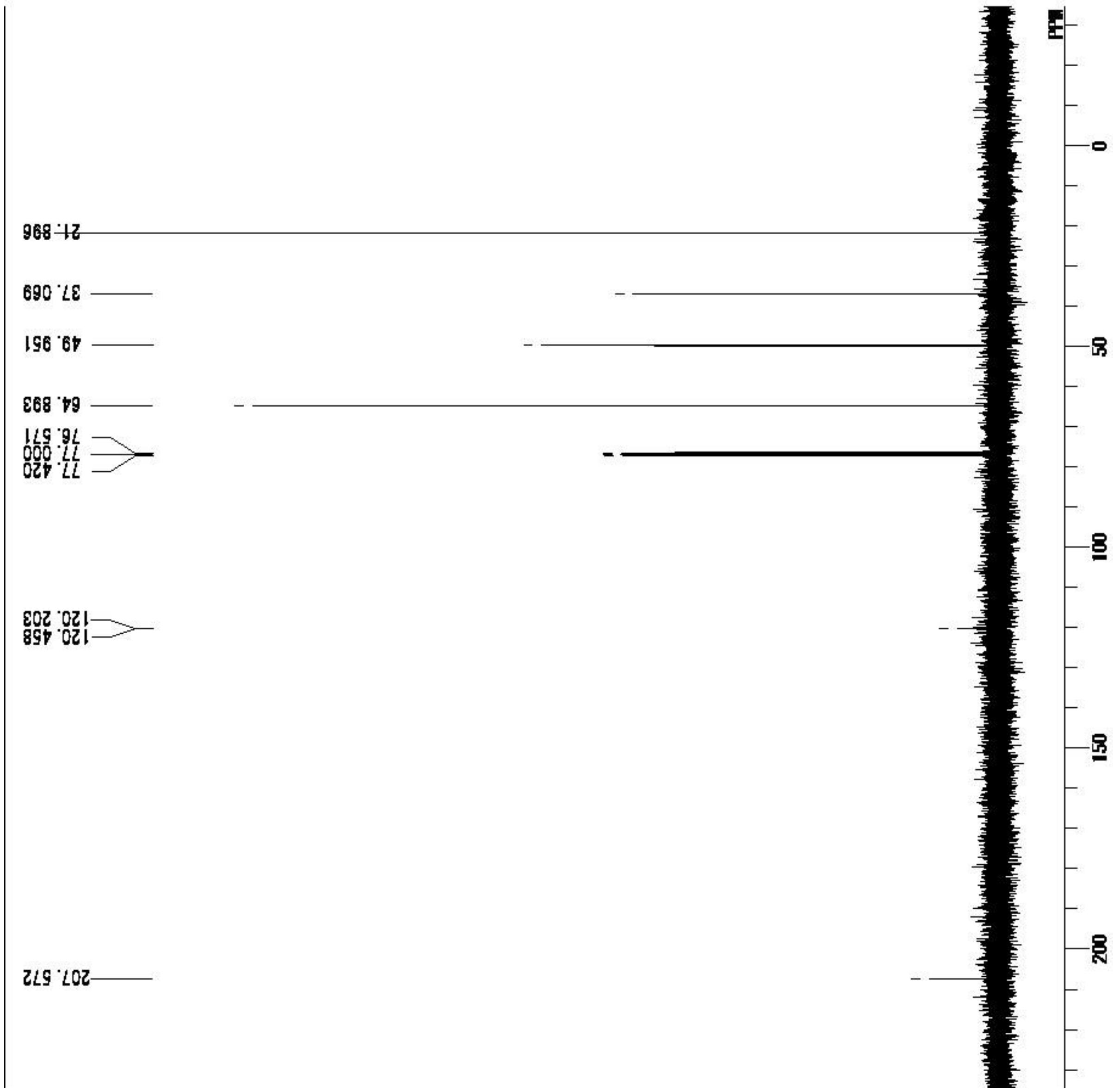

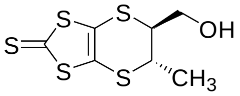

9

Figure S3-2: ^{13}C NMR (75 MHz, $CDCl_3$) spectrum of compound **9**.

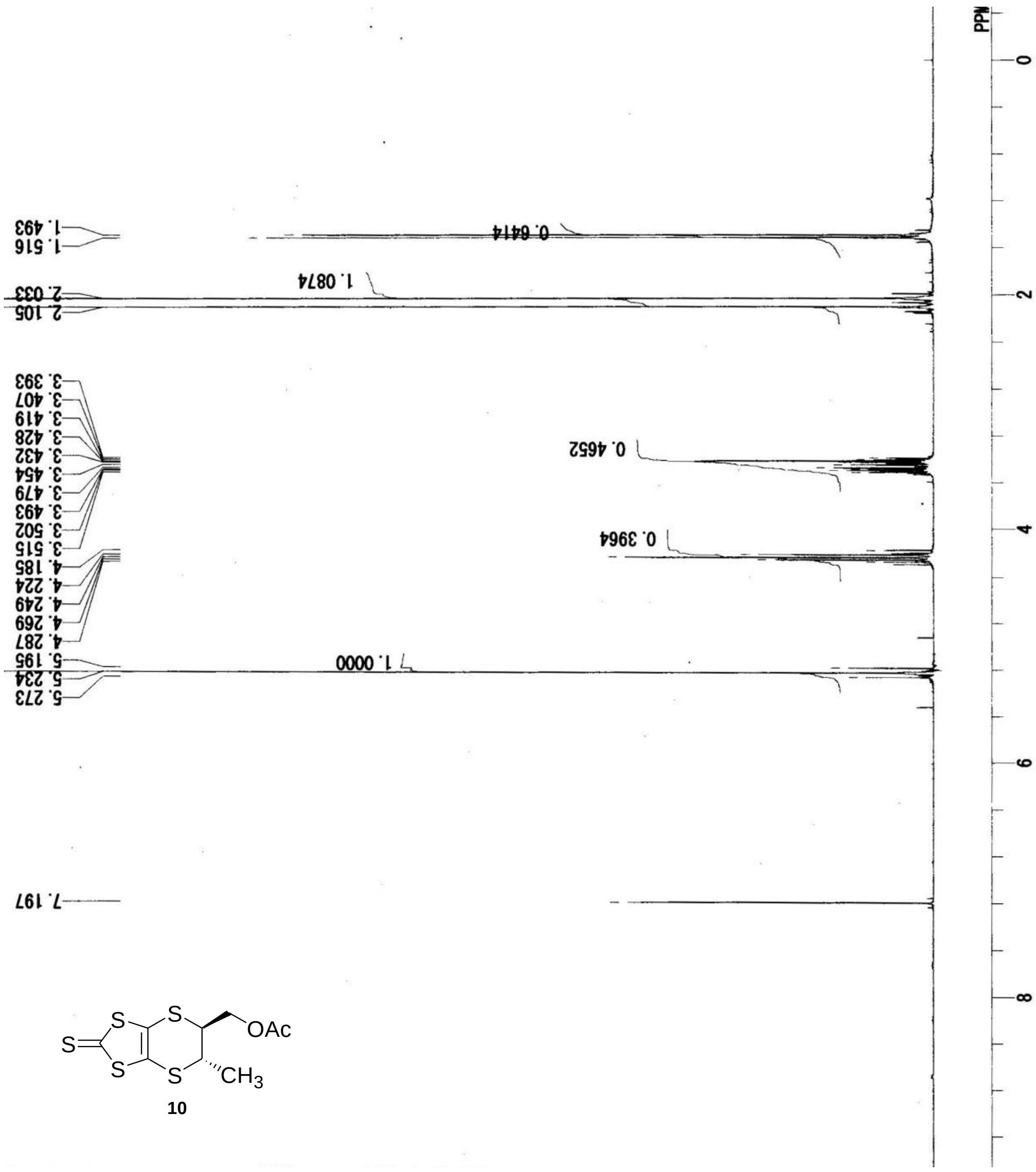

Figure S3-3: ^{1}H NMR (300 MHz, $CDCl_3$) spectrum of compound **10**.

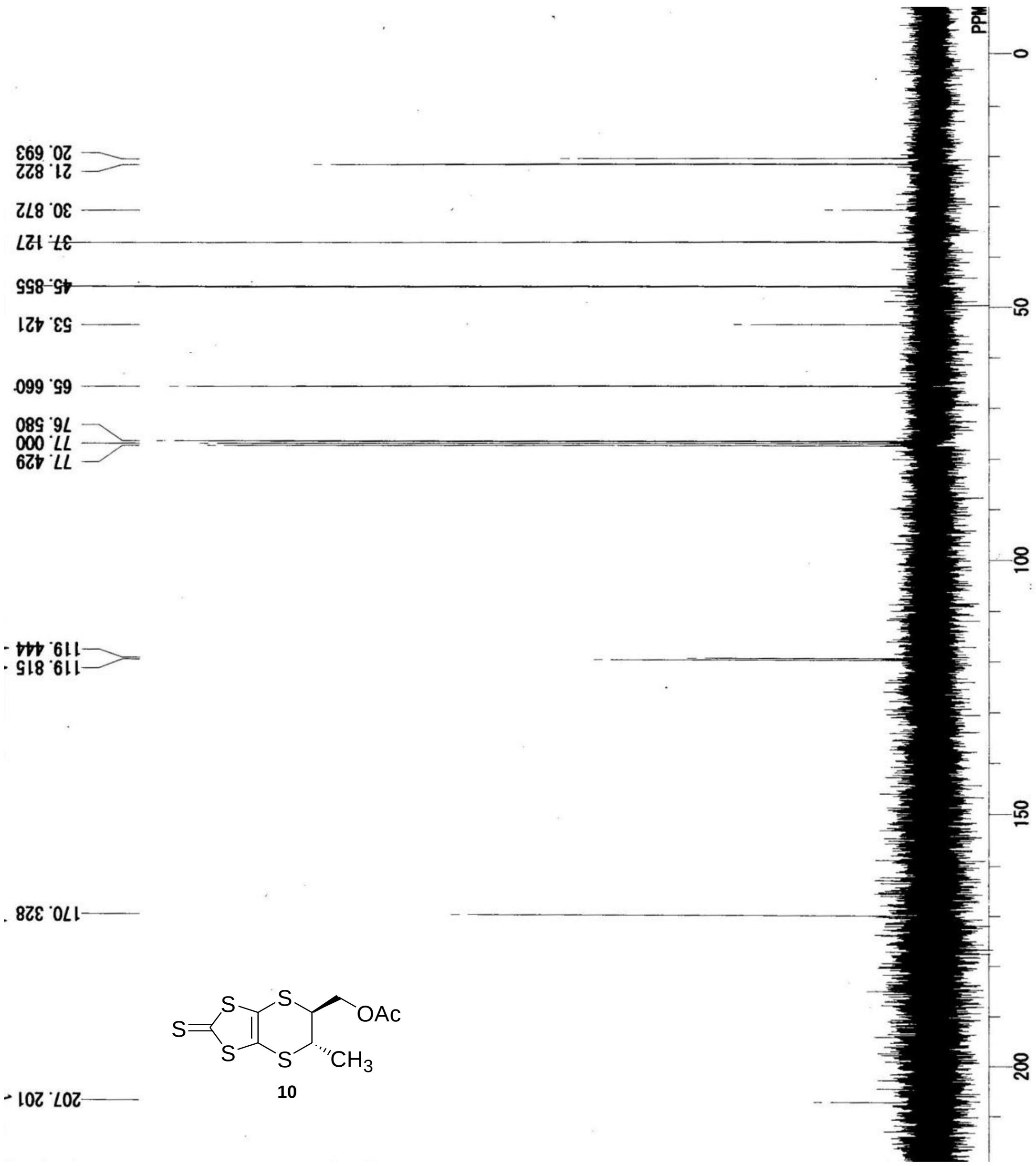

Figure S3-4: ^{13}C NMR (75 MHz, $CDCl_3$) spectrum of compound **10**.

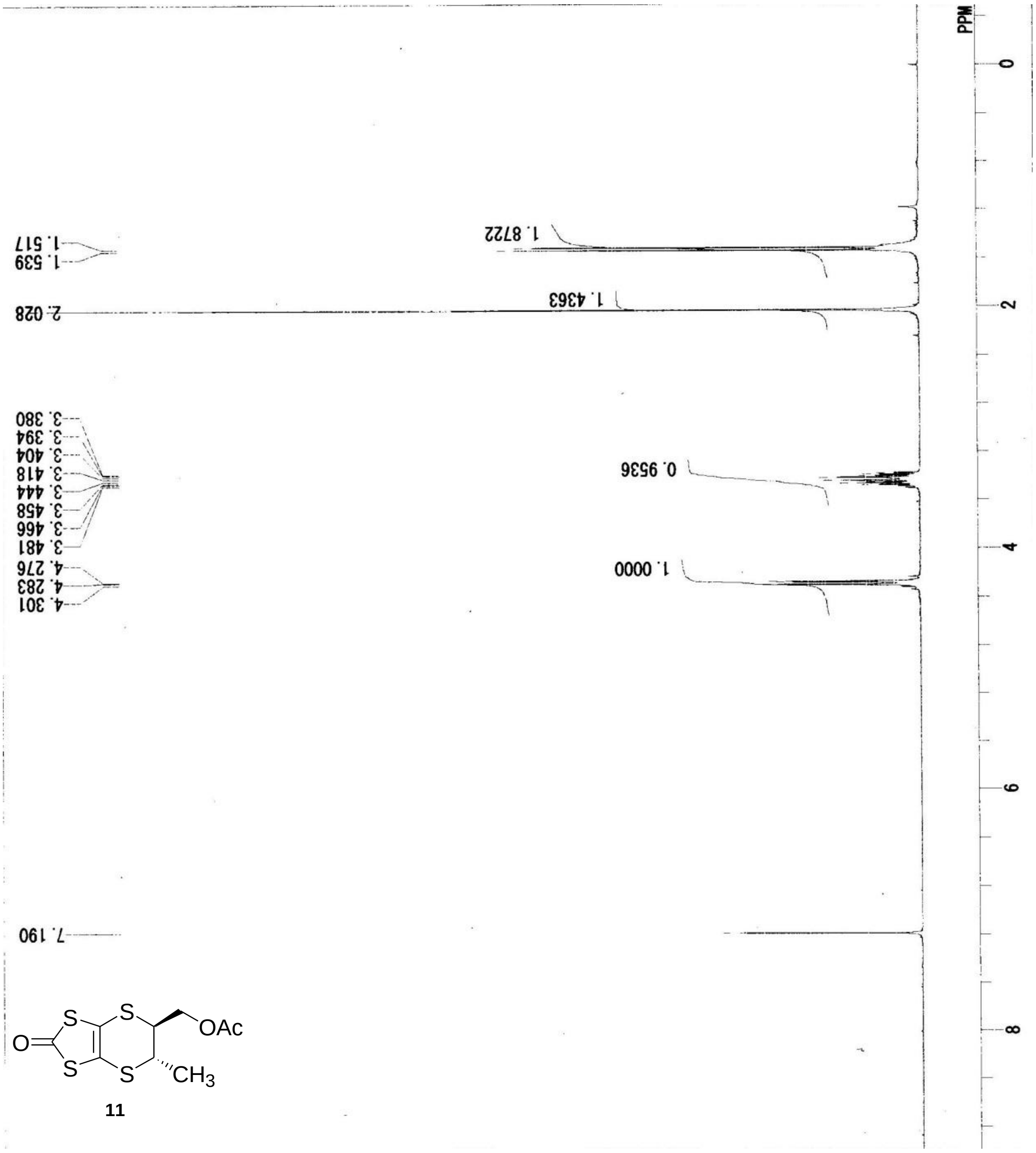

Figure S3-5: ^{1}H NMR (300 MHz, $CDCl_3$) spectrum of compound **11**.

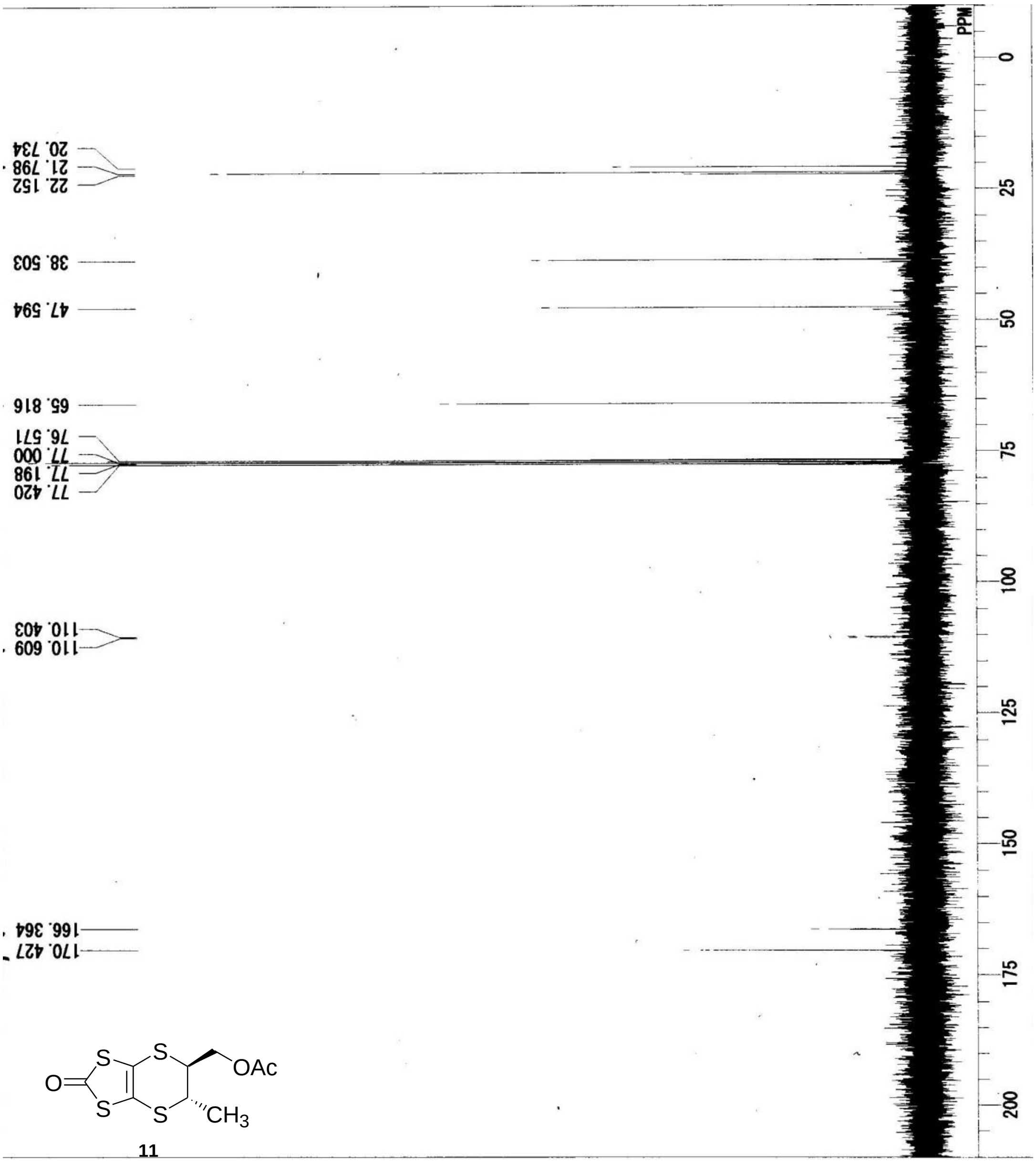

Figure S3-6: ^{13}C NMR (75 MHz, $CDCl_3$) spectrum of compound **11**.

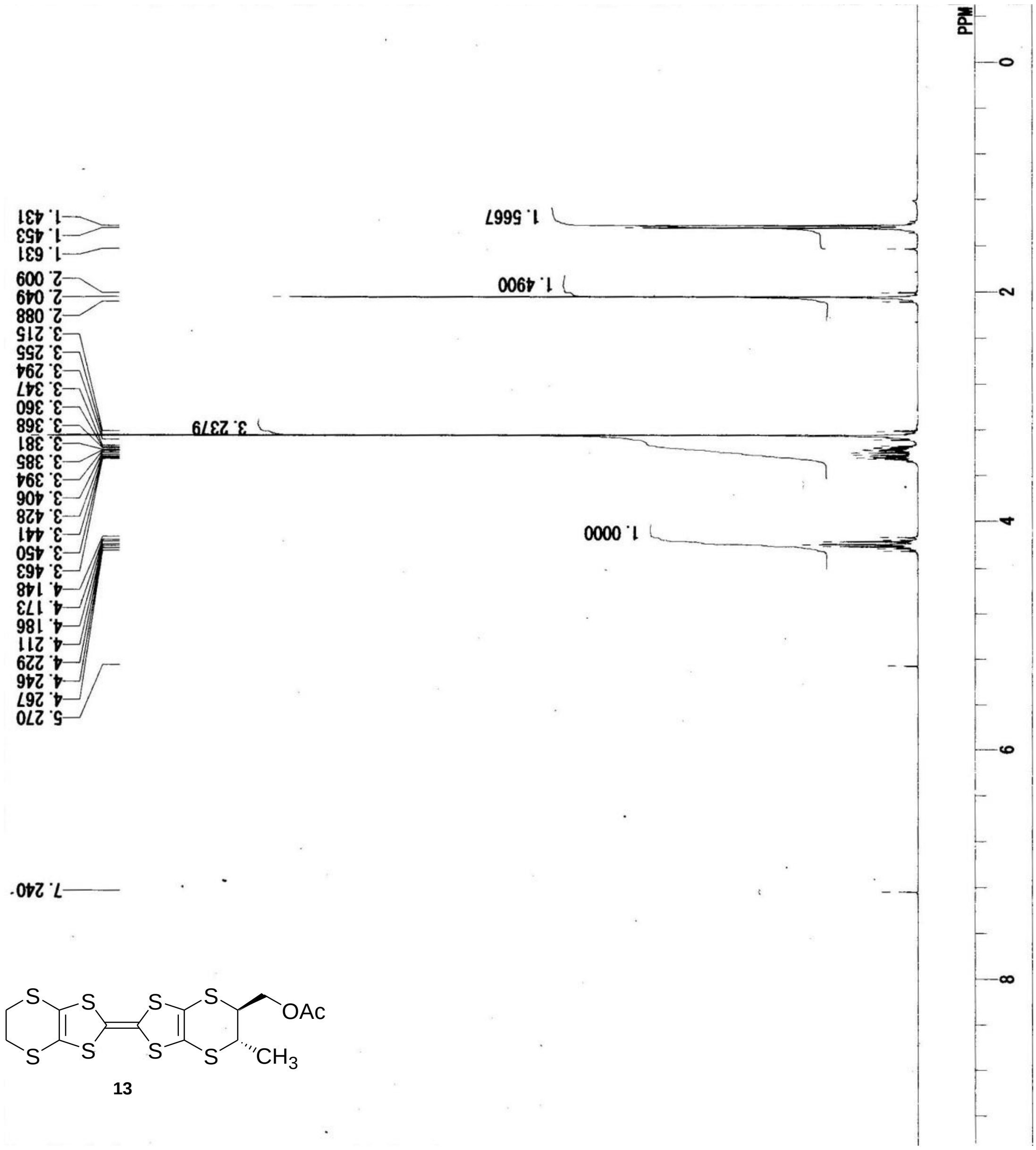

13

Figure S3-7: ^{1}H NMR (300 MHz, $CDCl_3$) spectrum of compound **13**.

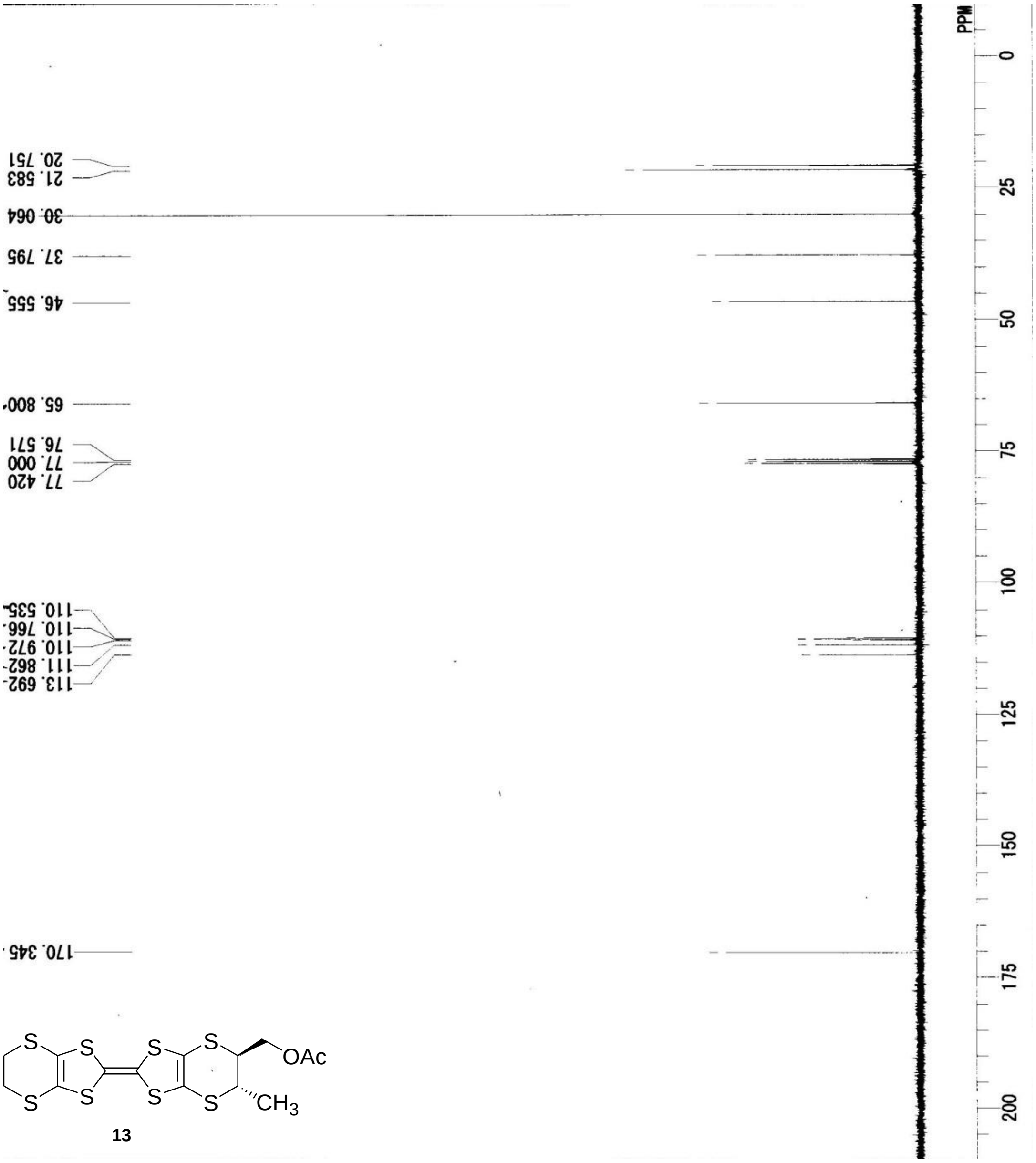

Figure S3-8: ^{13}C NMR (75 MHz, $CDCl_3$) spectrum of compound **13**.

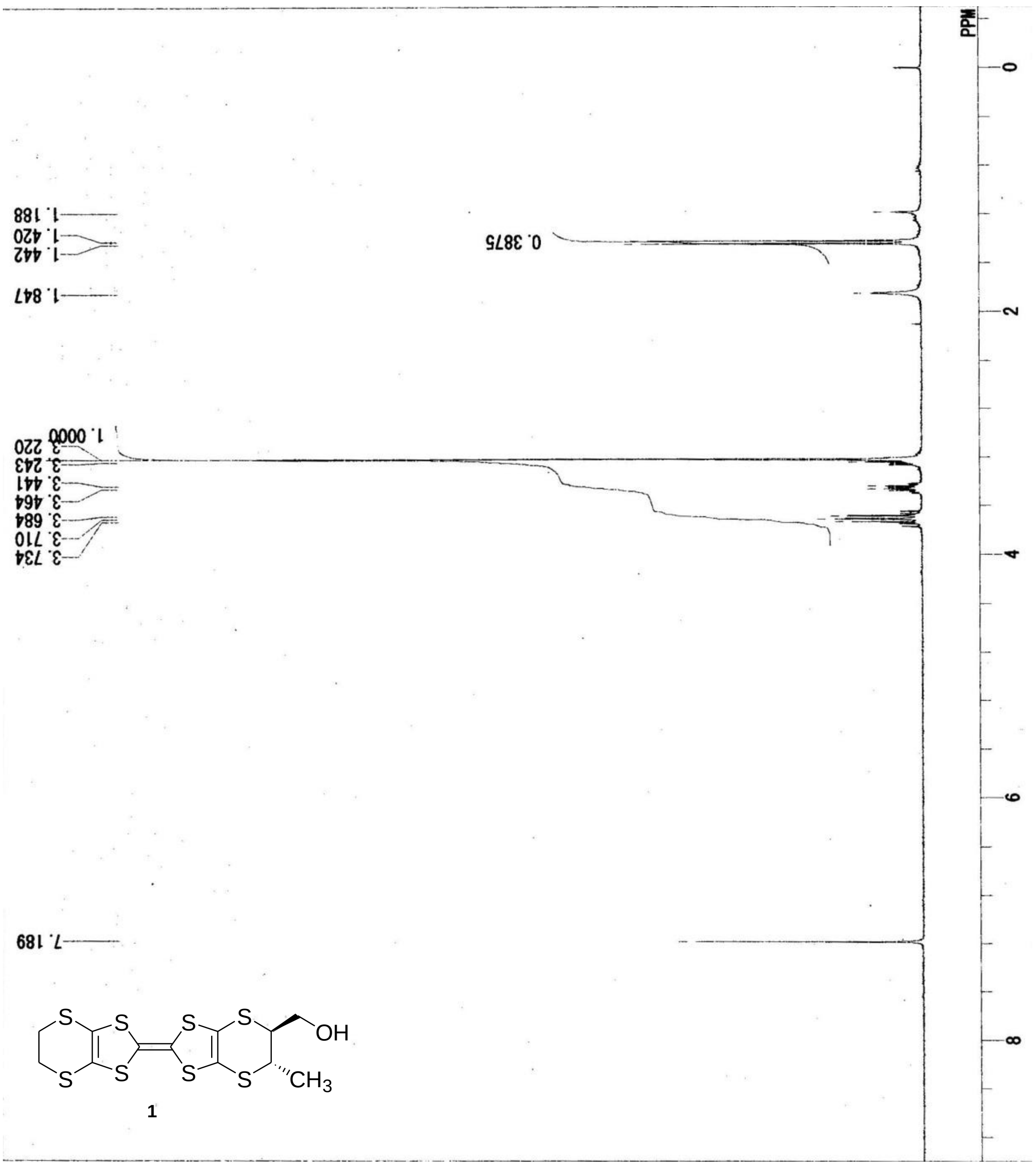

Figure S3-9: ^{1}H NMR (300 MHz, $CDCl_3$) spectrum of compound **1**.

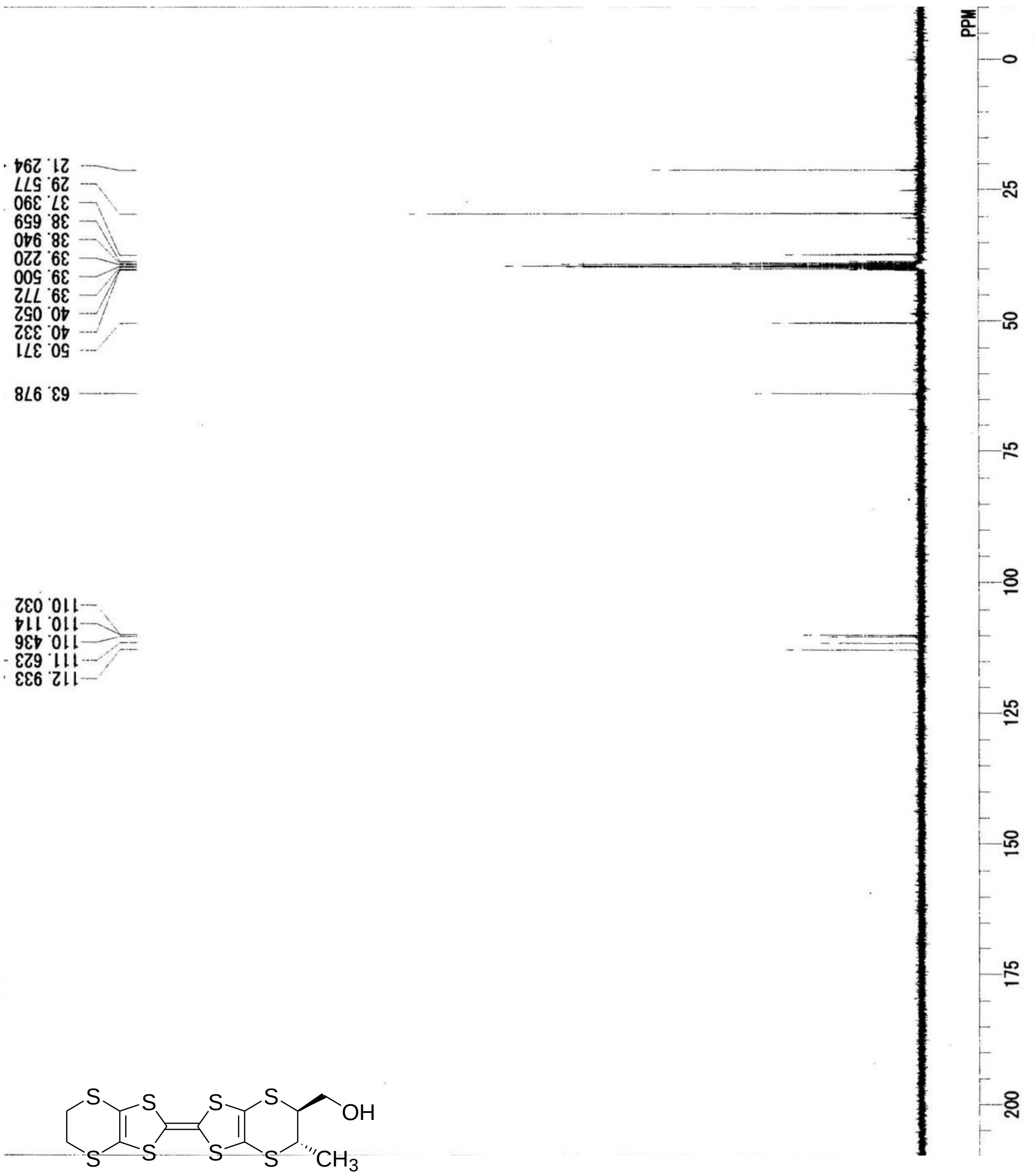

1

Figure S3-10: ^{13}C NMR (75 MHz, $CDCl_3$) spectrum of compound **1**.

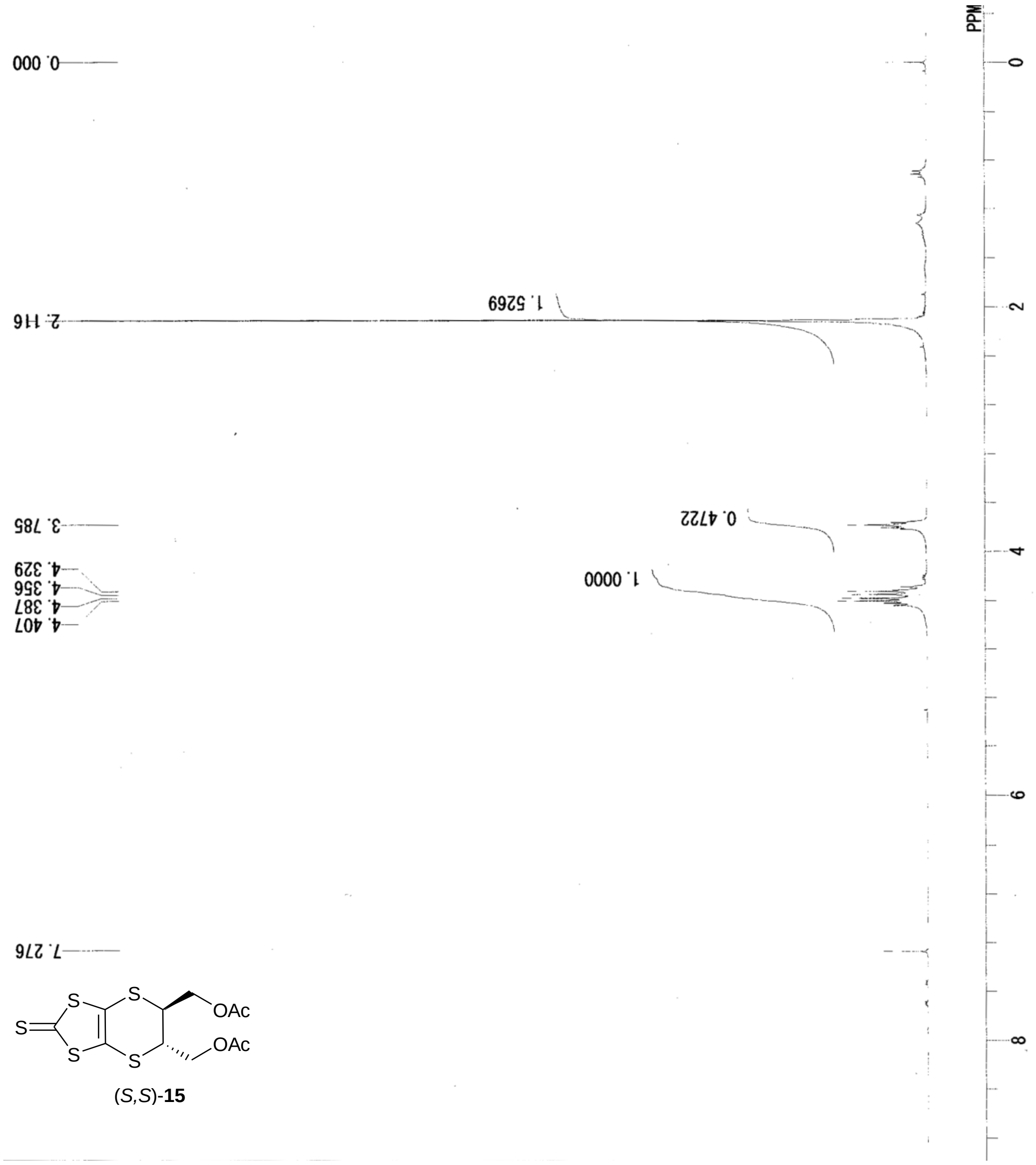

Figure S3-11: ^{1}H NMR (300 MHz, $CDCl_3$) spectrum of compound **15**.

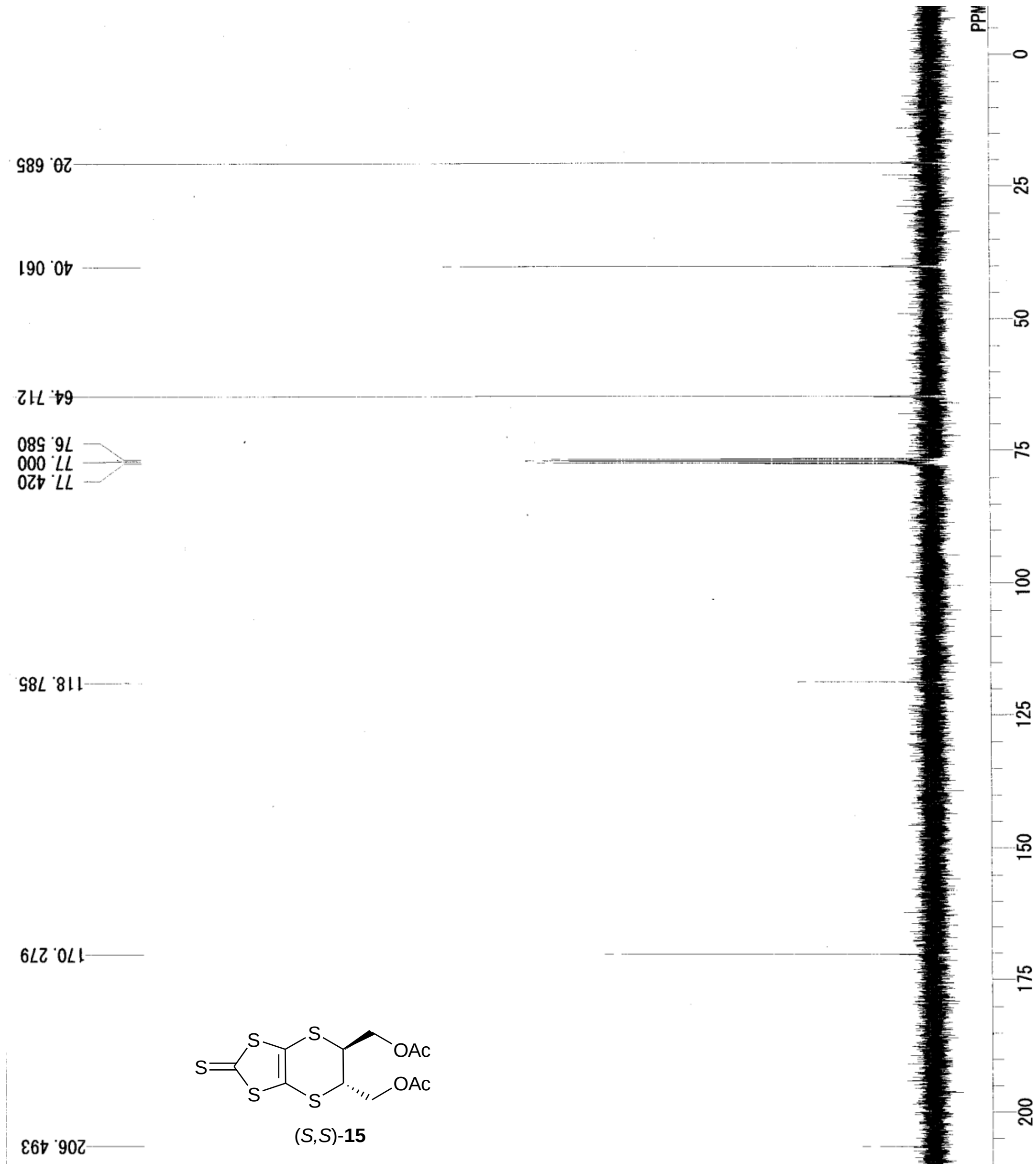

Figure S3-12: ^{13}C NMR (75 MHz, $CDCl_3$) spectrum of compound **15**.

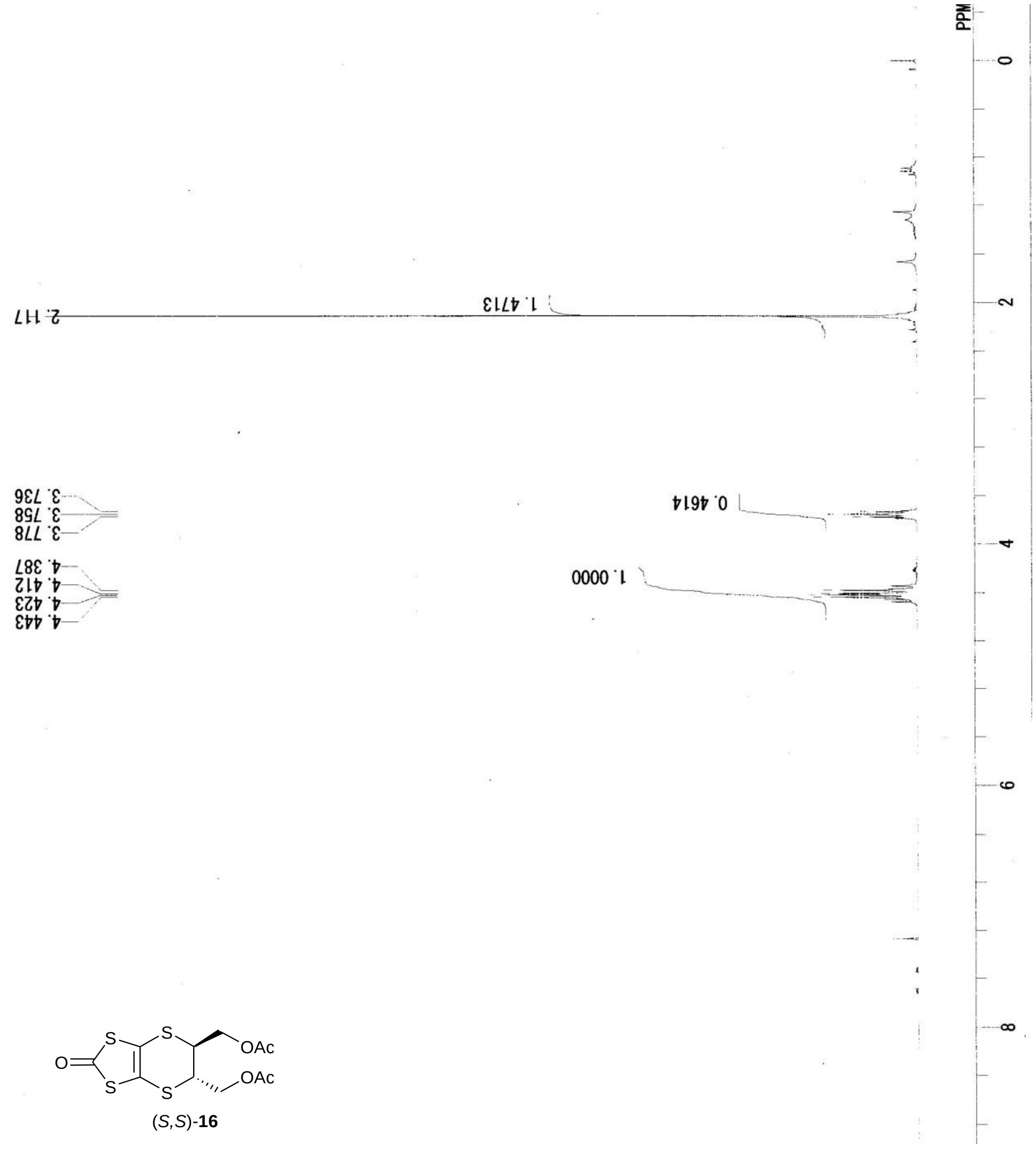

Figure S3-13: ^{1}H NMR (300 MHz, $CDCl_3$) spectrum of compound **16**.

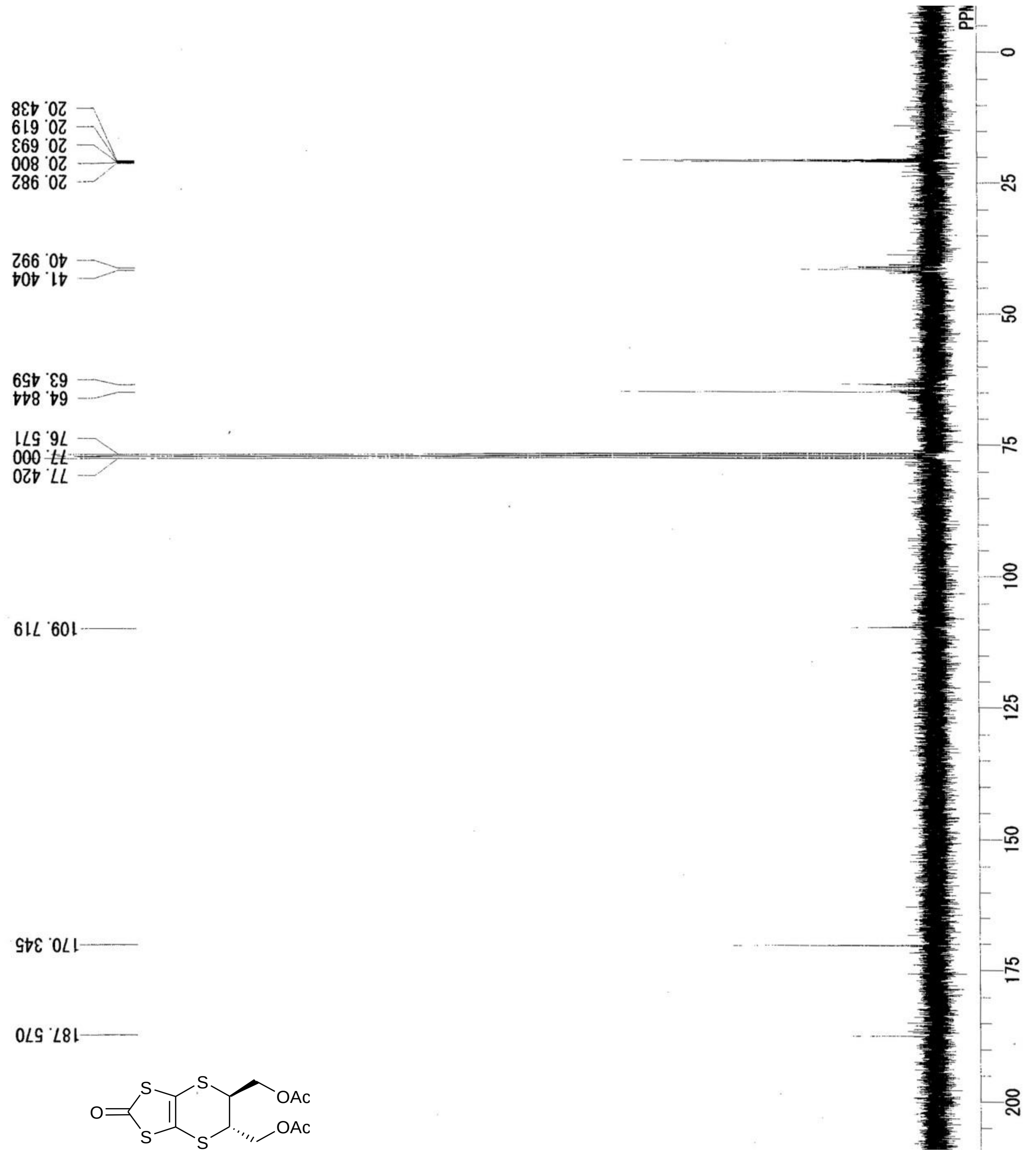

(*S*,*S*)-**16**

Figure S3-14: ^{13}C NMR (75 MHz, $CDCl_3$) spectrum of compound **16**.

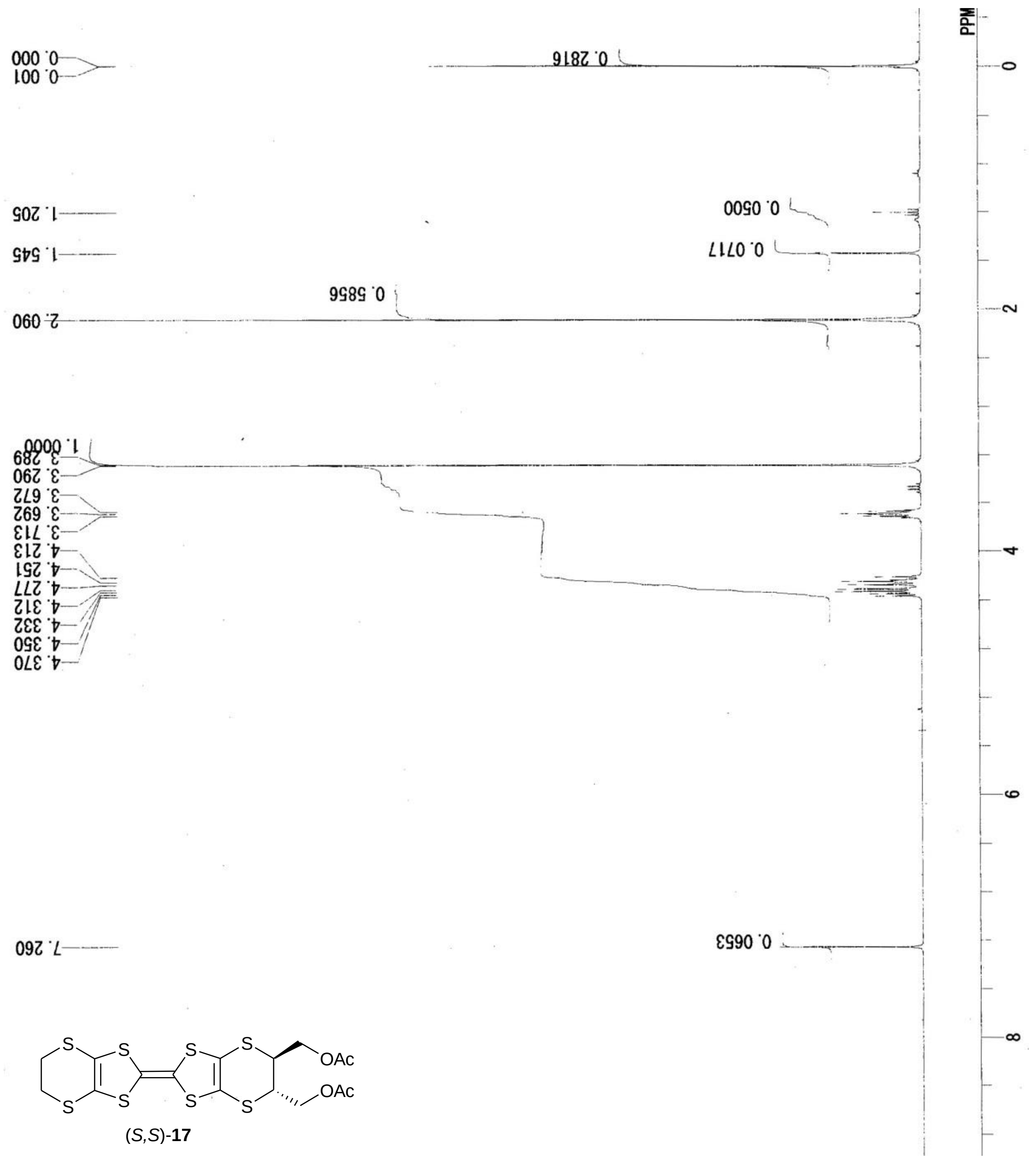

Figure S3-15: ^{1}H NMR (300 MHz, $CDCl_3$) spectrum of compound **17**.

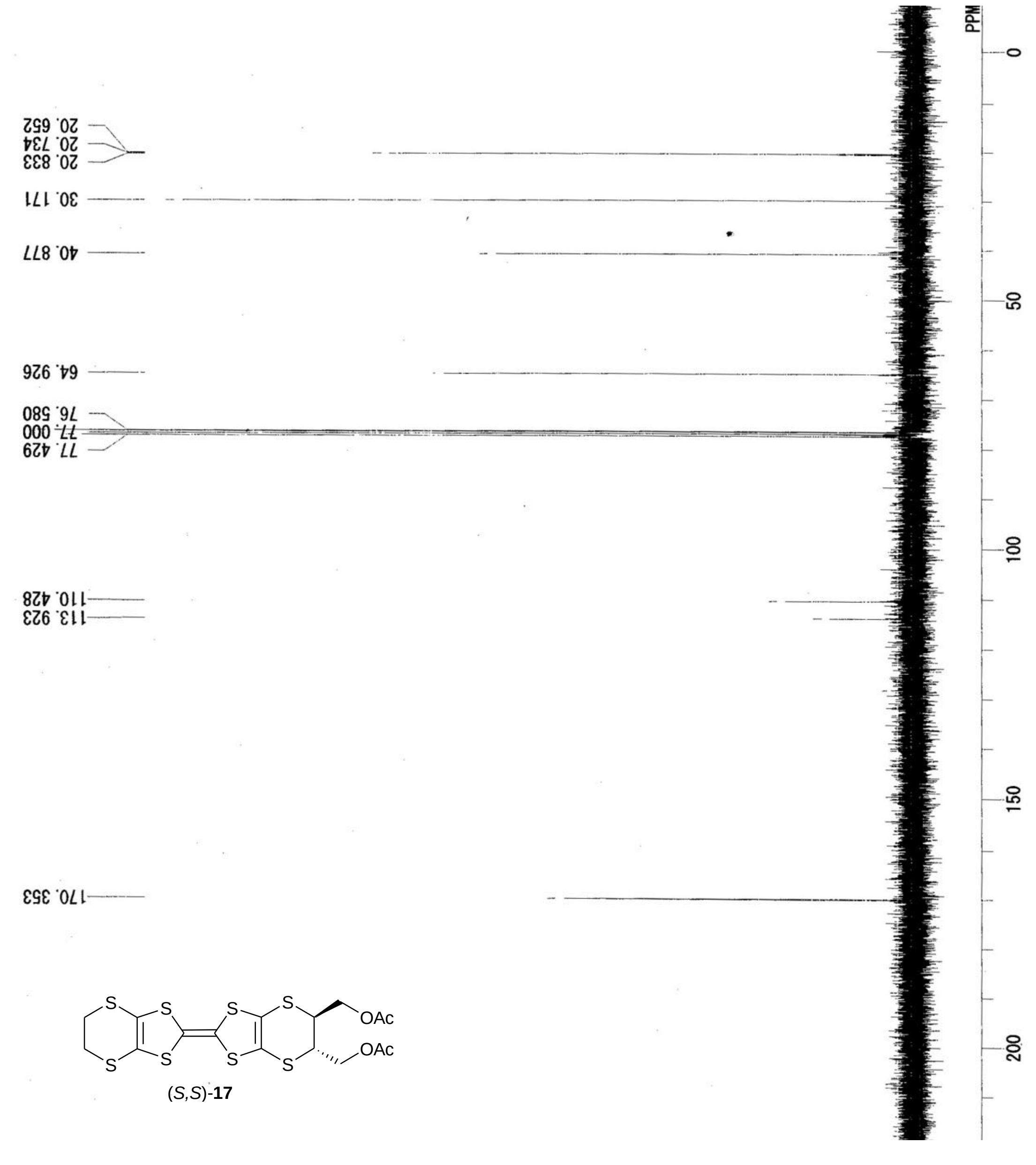

Figure S3-16: ^{13}C NMR (75 MHz, $CDCl_3$) spectrum of compound **17**.

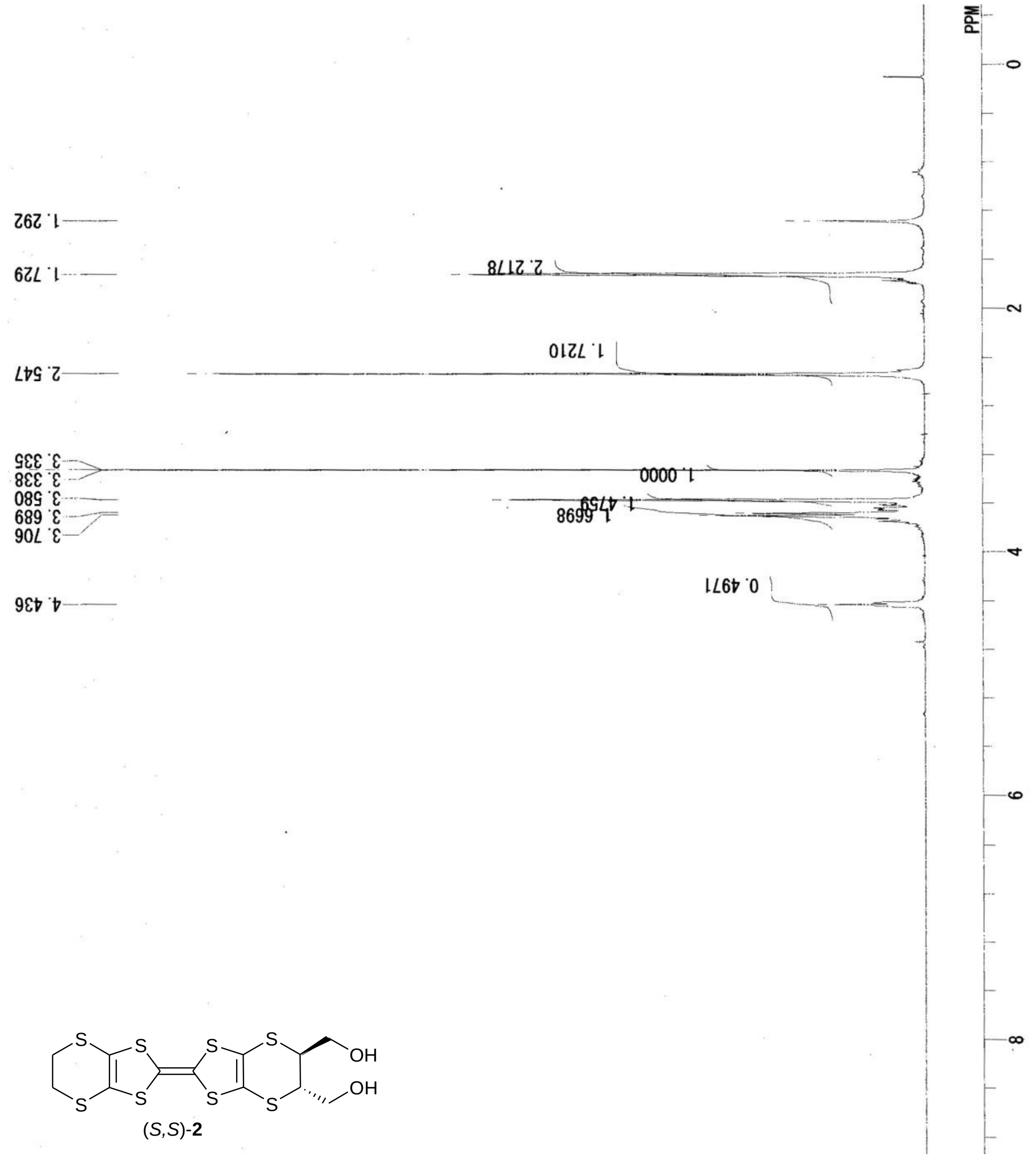

Figure S3-17: ^{1}H NMR (300 MHz, $CDCl_3$) spectrum of compound (*S,S*)-**2**.

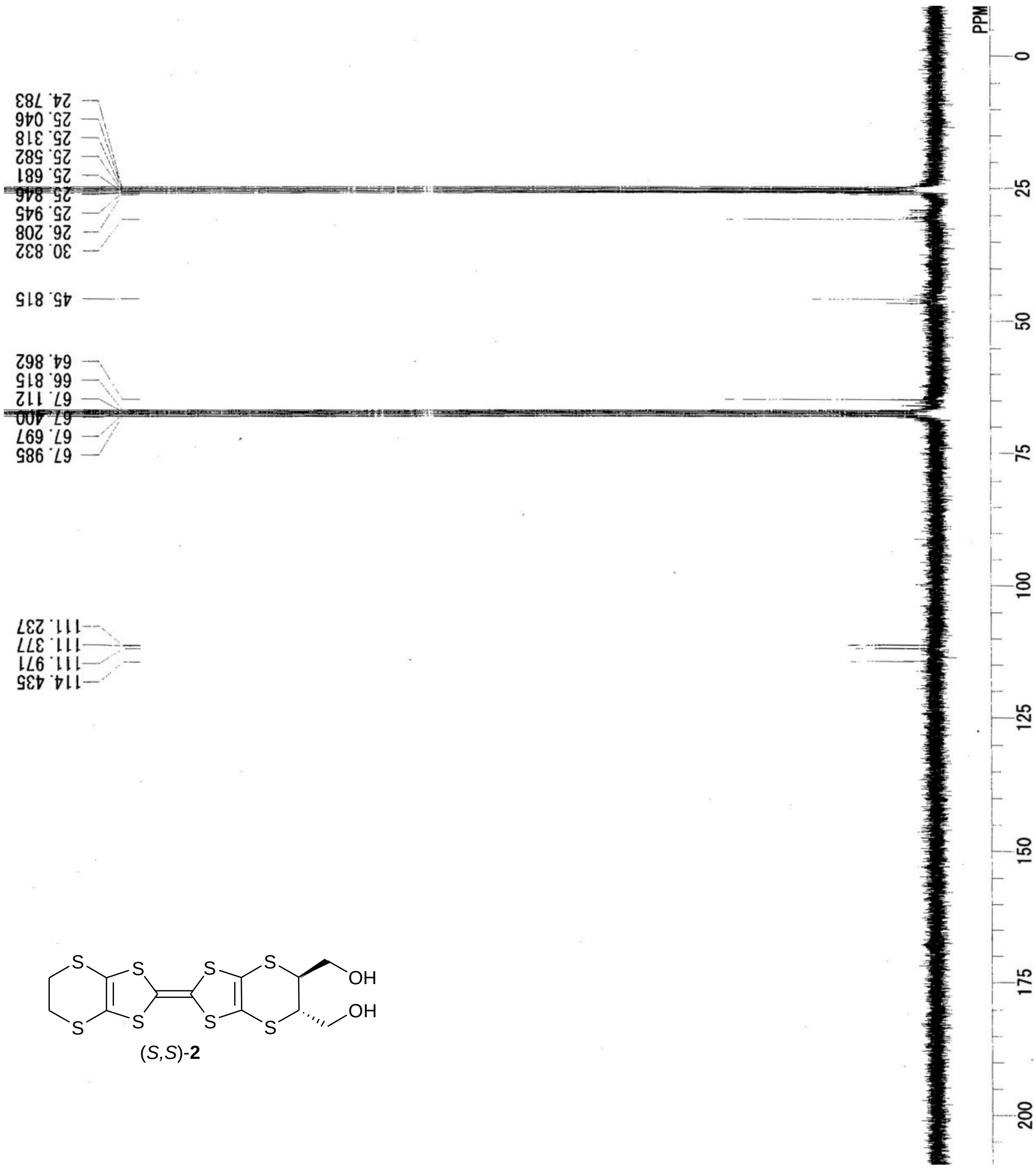

Figure S3-18: ^{13}C NMR (75 MHz, $CDCl_3$) spectrum of compound (*S*,*S*)-**2**.

References

S1 Krivickas, S. J.; Hashimoto, C; Takahashi, K.; Wallis, J. D.; Mori, H. *Phys. Stat. Solidi C* **2012**, *9*, 1146-1148.

S2 Guionneau, P.; Kepert, C. J.; Bravic, G.; Chasseau, D.; Truter, M. R.; Kurmoo, M.; Day, P. *Synthetic Metals* **1997**, *86*, 1973-1974.
